# Supplementary material for: Structural diversity of axonemes across mammalian motile cilia
Source: Nature. 2025 Jan 1;637(8048):1170–7. doi: 10.1038/s41586-024-08337-5 (PMC11779644; doi:10.1038/s41586-024-08337-5)
Supplement: Supplementary file 1 — This file contains Supplementary Figs. 1–33, Tables 1–3 and 5 and references. [file 41586_2024_8337_MOESM1_ESM.docx]

**
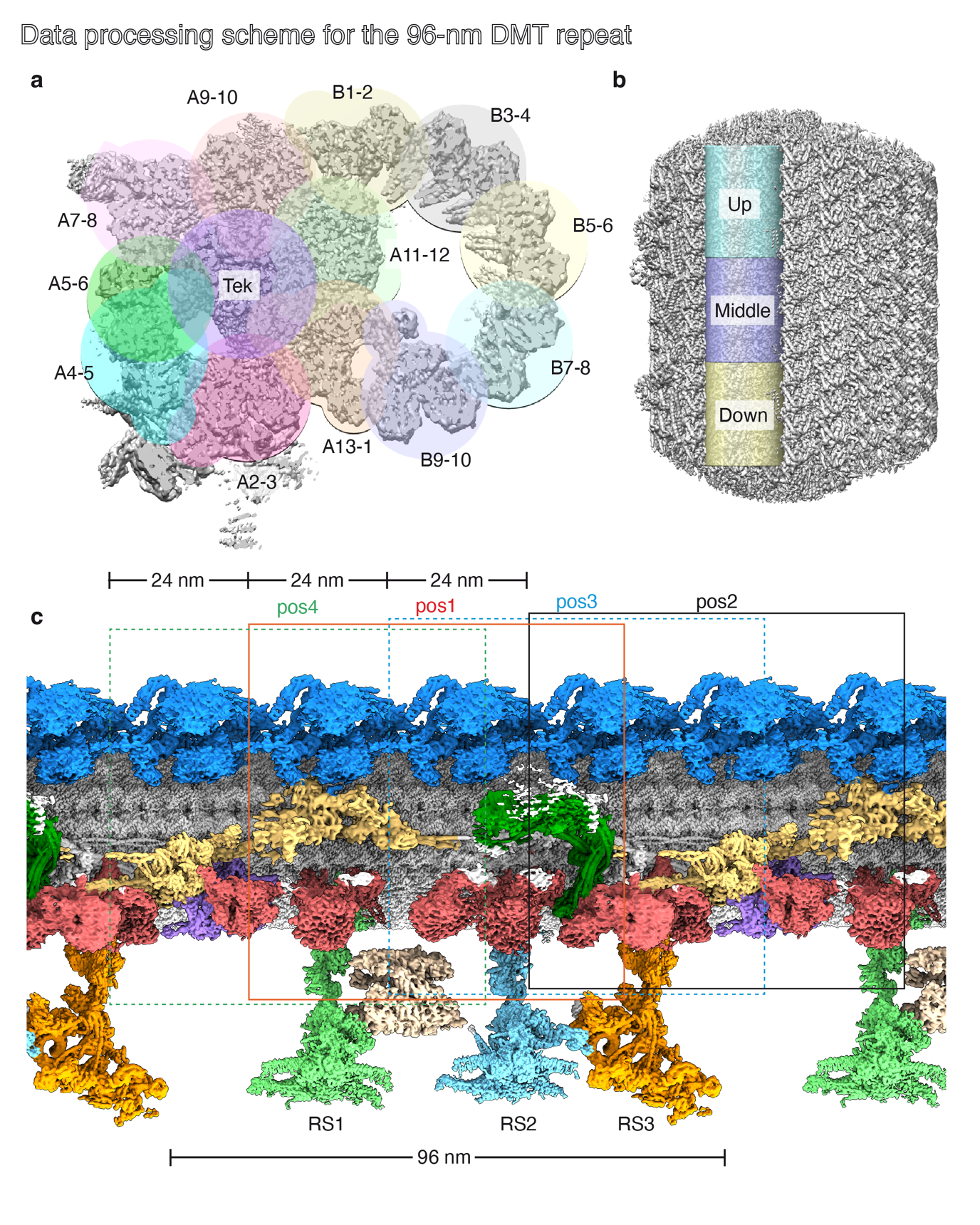
Supplementary Figure 1 | Cryo-EM single particle analysis processing scheme for the 96-nm repeat of the axonemal doublet microtubule from bovine sperm. (a)** A set of cylindrical masks were used to divide the DMT radially into 13 sub-regions. **(b)** Each cylindrical mask was further divided into 3 shorter segments (up, middle and down). Consequently, the entire DMT structure was divided into a total of 39 local regions. **(c)** Due to computational constraints, we used a box size of 512 pixels (666 Å) for 3D reconstruction. As a result, we used 4 different reconstruction boxes (positions 1-4) to cover the 96-nm repeat length. The centers of each box were 24-nm apart.


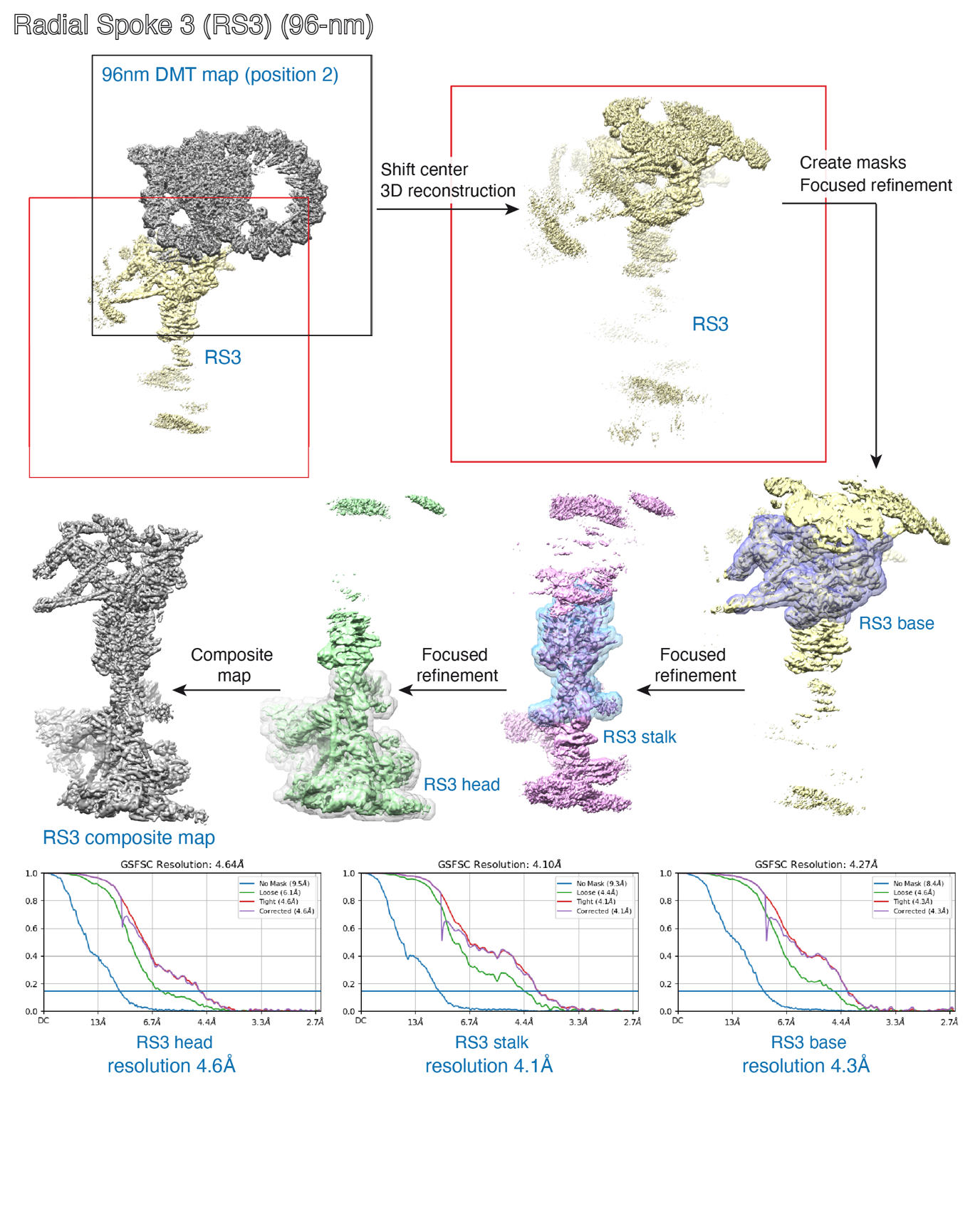


**Supplementary Figure 2 | Processing scheme for radial spoke 3 (RS3) in bovine sperm.** The reconstruction box “position 2” was used as the starting point to calculate a map around RS3 (see also Supplementary Figure 1). After shifting the center of the box to RS3, iterative local refinements were performed starting with a mask around the RS3 base, followed by the stalk, and finally the head. Maps were then aligned and merged into a composite map using *vop maximum* in Chimera. Similar processing workflows were applied to RS1 and RS2 in sperm and other cell types.

**
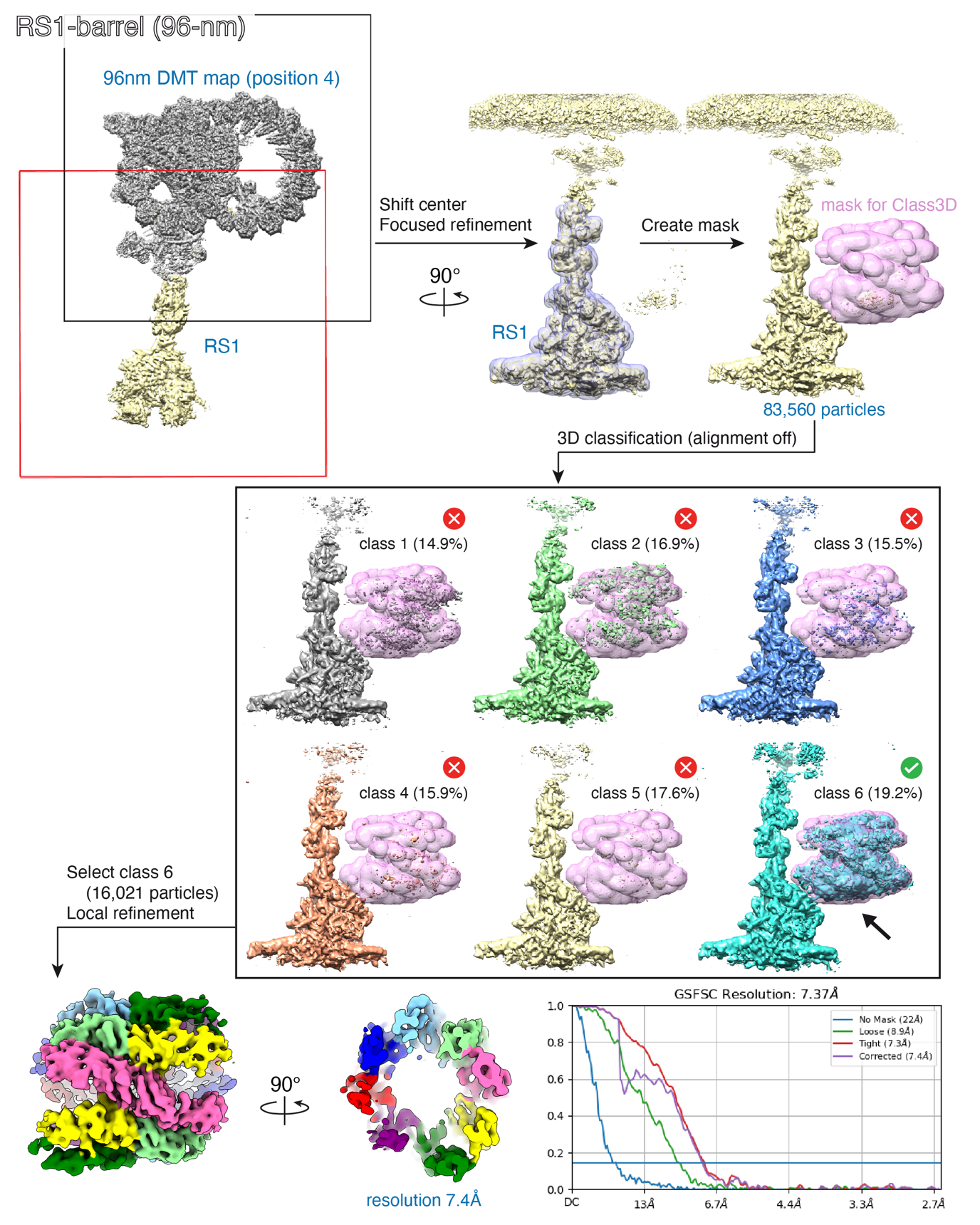
**

**Supplementary Figure 3 | Processing scheme for the RS1-associated barrel in bovine sperm.** The reconstruction box “position 4” was used as the starting point to calculate a map around RS1 (see also Supplementary Figure 1). The center of the box was shifted to RS1, followed by a local refinement around the RS1 head. At this point, density corresponding to the barrel was visible. 3D classification without alignment was performed using a custom mask in the shape of the barrel (derived from a previous cryo-ET map EMD-27453 ^1^). Particles belonging to the class with strongest density for the barrel (~19% of particles) were then locally refined.

**
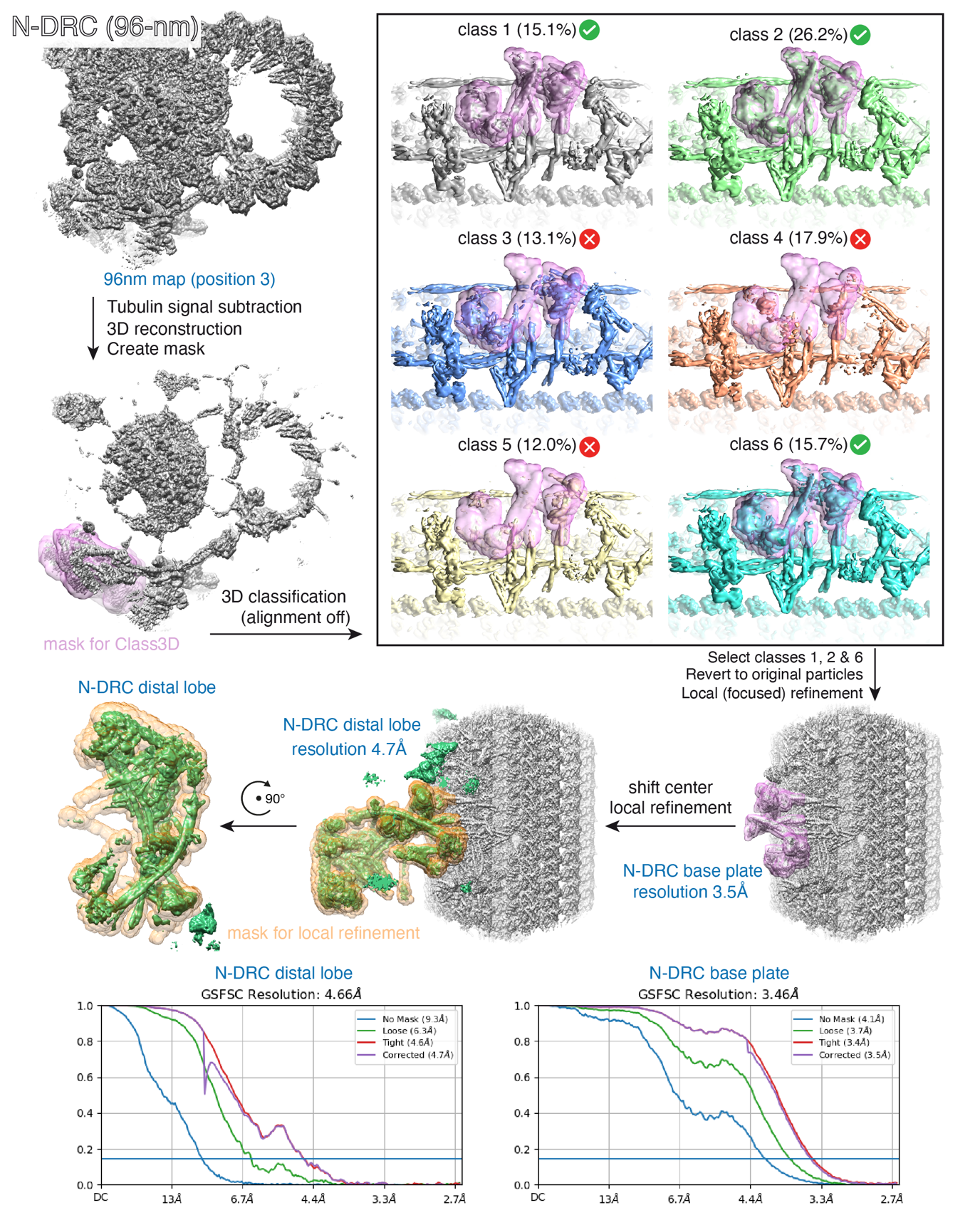
**

**Supplementary Figure 4 | Processing scheme for the nexin-dynein regulatory complex (N-DRC) in bovine sperm.** The reconstruction box “position 3” was used as the starting point to calculate a map around the N-DRC (see also Supplementary Figure 1). Tubulin signal was subtracted and 3D classification performed using a mask around the N-DRC baseplate. Classes showing clear density were pooled, reverted to original particles, and locally refined. The box was then shifted towards the N-DRC distal lobe, followed by local refinement with a corresponding mask. An identical workflow was used for all cilium types in this study.

**
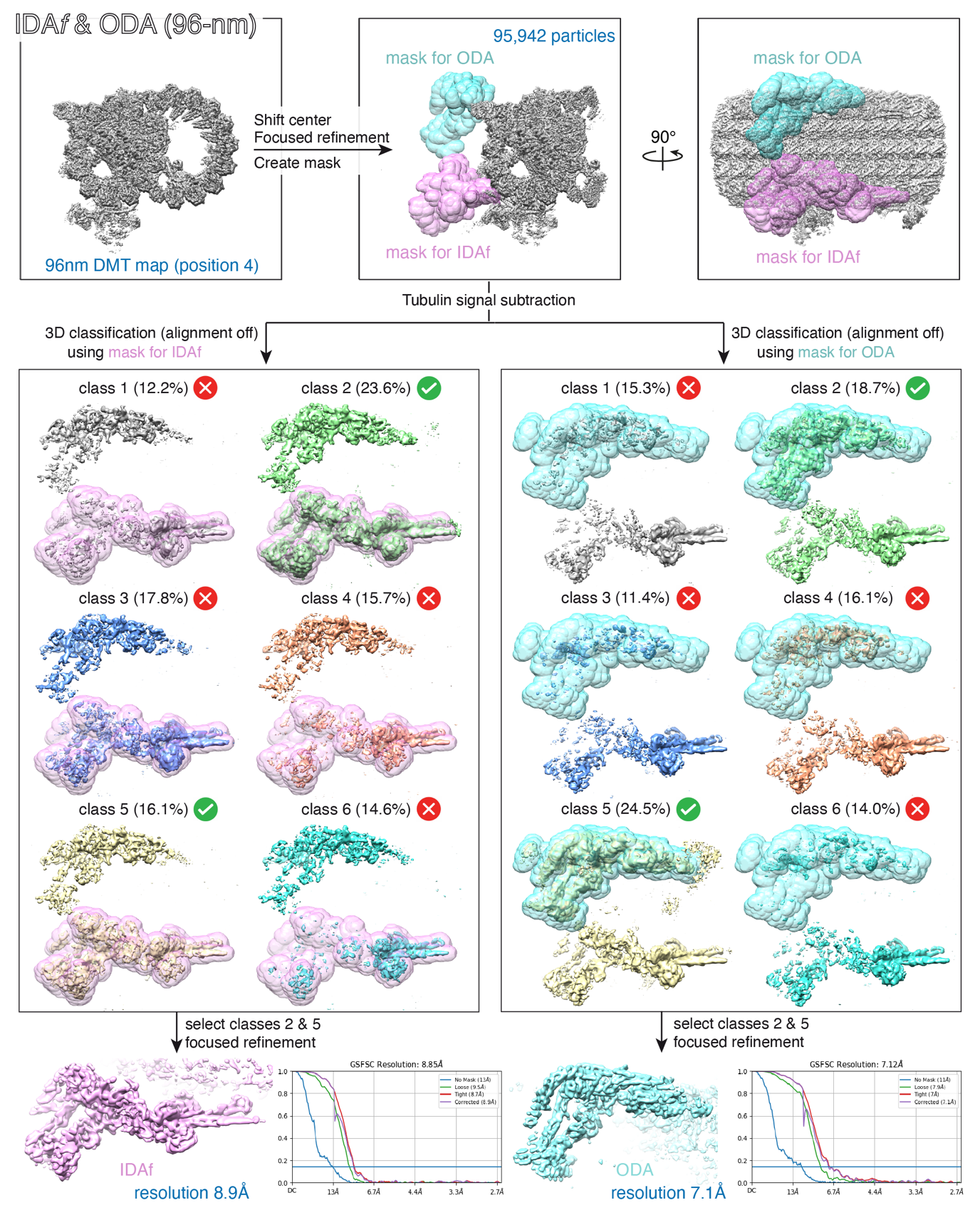
**

**Supplementary Figure 5 | Processing scheme for the intermediate chain/light chain (IC/LC) complexes of IDA*f* and its adjacent ODA in bovine sperm.** The reconstruction box “position 4” was used as the starting point to calculate a map around IDA*f* (see also Supplementary Figure 1). After centering the box between the IDA*f* IC/LC complex and its neighboring ODA, tubulin signal was subtracted and independent 3D classifications without alignment were performed using masks around either IDA*f* or ODA. Good classes were pooled and subjected to local refinements to generate the final maps. An identical workflow was used for all cilium types in this study.

**
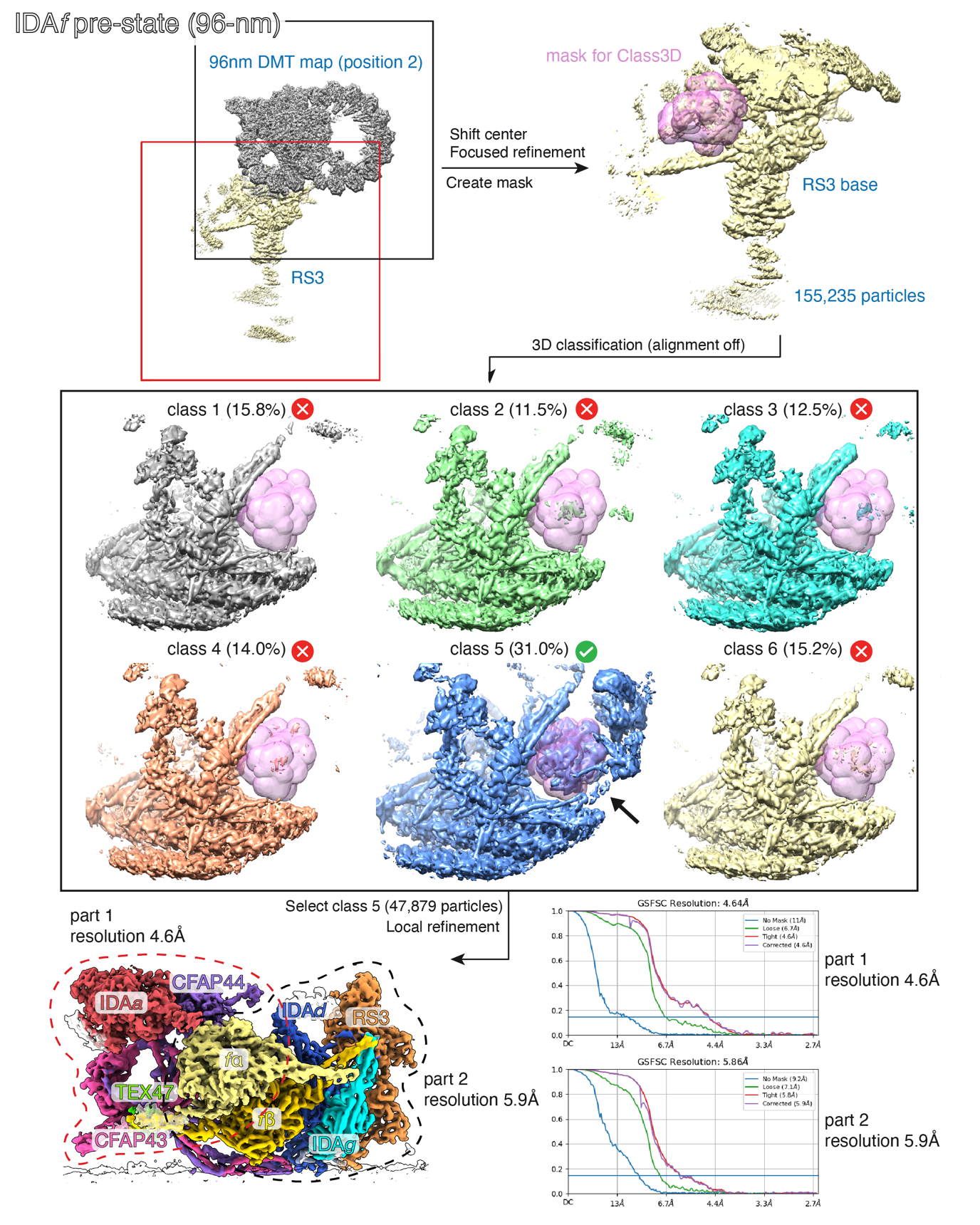
**

**Supplementary Figure 6 | Processing scheme for the IDA*f* pre-stroke state in bovine sperm.** The reconstruction box “position 2” was used as the starting point to calculate a map around RS3 (see also Supplementary Figure 1). A local refinement was performed around the RS3 base, followed by no-alignment 3D classification using a mask around the IDA*fβ* motor domain visible below IDA*d/g*. The class with strongest density (~31% of particles) was selected for subsequent focused refinements of IDA*f* and neighboring complexes. An identical workflow was used for all cilium types in this study.

**
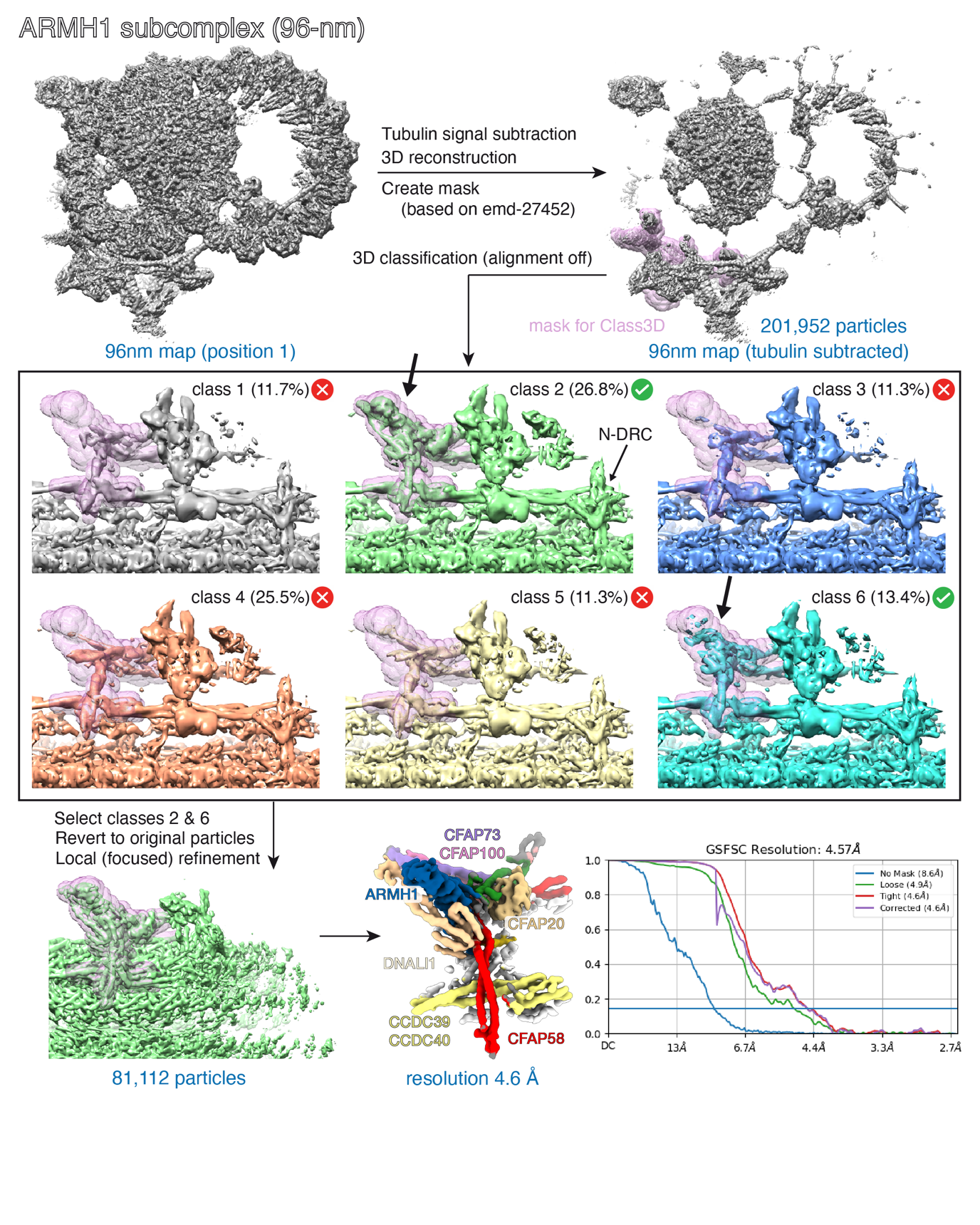
**

**Supplementary Figure 7 | Processing scheme for the ARMH1 subcomplex in bovine sperm.** The reconstruction box “position 1” was used as the starting point to calculate a map around the bases of RS1, RS2, and IDA*c* (see also Supplementary Figure 1). In the initial consensus maps, weak density was visible atop the CFAP58 dimer that was not accounted for in available models. After tubulin signal subtraction, 3D classification was performed using a mask derived from previous cryo-ET map (EMD-27452 ^1^), which allowed recovery of classes enriched for the density. Subtracted particles were then reverted to original particles and local refinements performed, which resulted in a 4.6 Å map that could be used to identify the density as ARMH1 (see also Data S1).

**
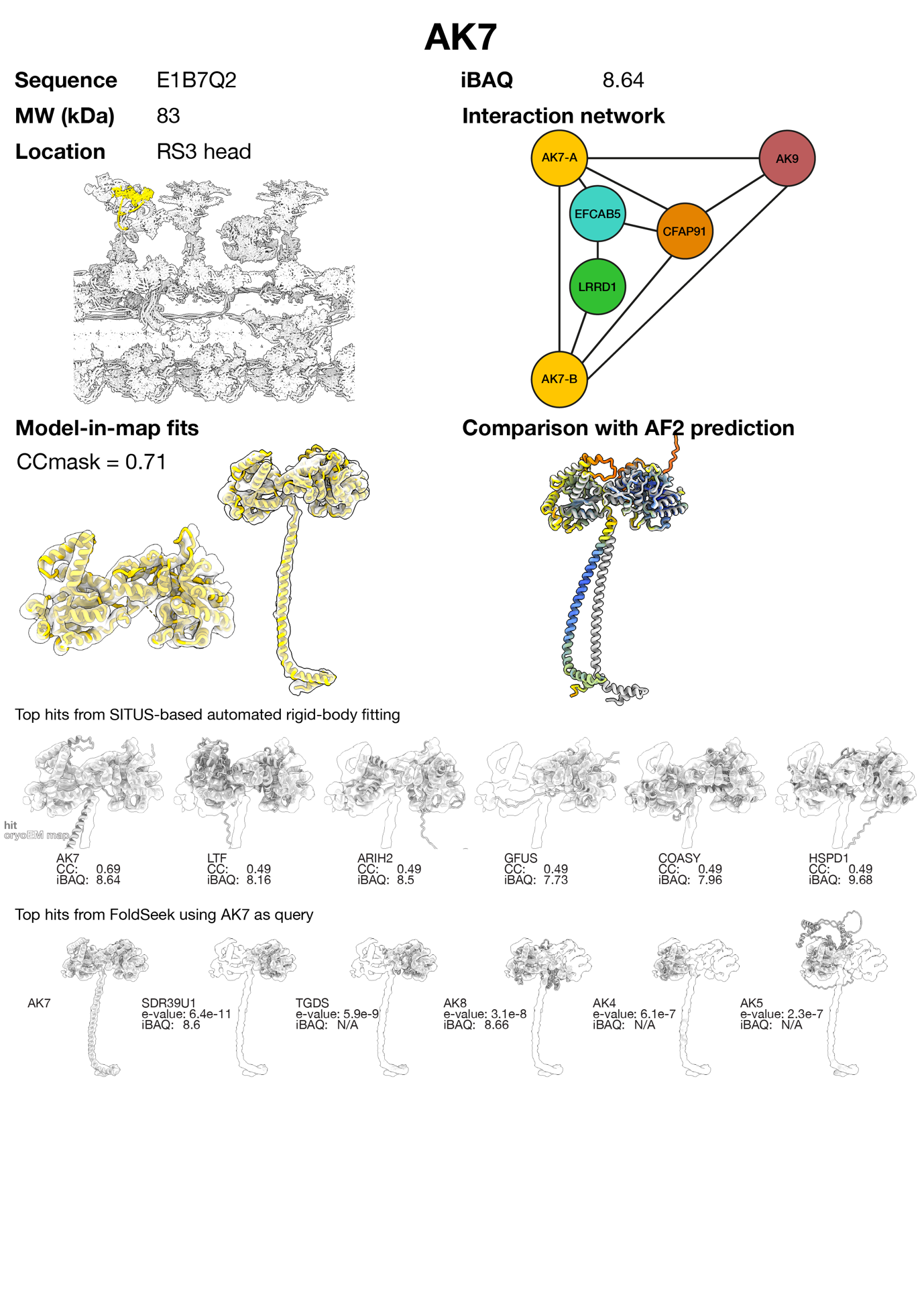
**

**Supplementary Figure 8 | Protein identification and assessment report for AK7.** AK7 was identified by SITUS-based automated rigid-body fitting of an AlphaFold library of the bovine sperm proteome. Other hits were excluded because they did not fit the density as well as AK7. To find alternatives with similar folds, AK7 was used as a query in FoldSeek to search the entire human AlphaFold database. Top hits from this approach were discarded because they did not match the density as well as AK7 and/or were not detected in the bovine sperm proteome. Supporting evidence: (i) immunofluorescence localizes AK7 along the human sperm flagellum^2^ and along axonemes of *Xenopus* multi-ciliated cells^3^, (ii) AK7 co-immunoprecipitates with the RS3 protein CFAP251 in *Tetrahymena*^4^, and (iii) AK7 levels are reduced when the RS3 protein CFAP91 is knocked-out in *Tetrahymena*^5^.

**
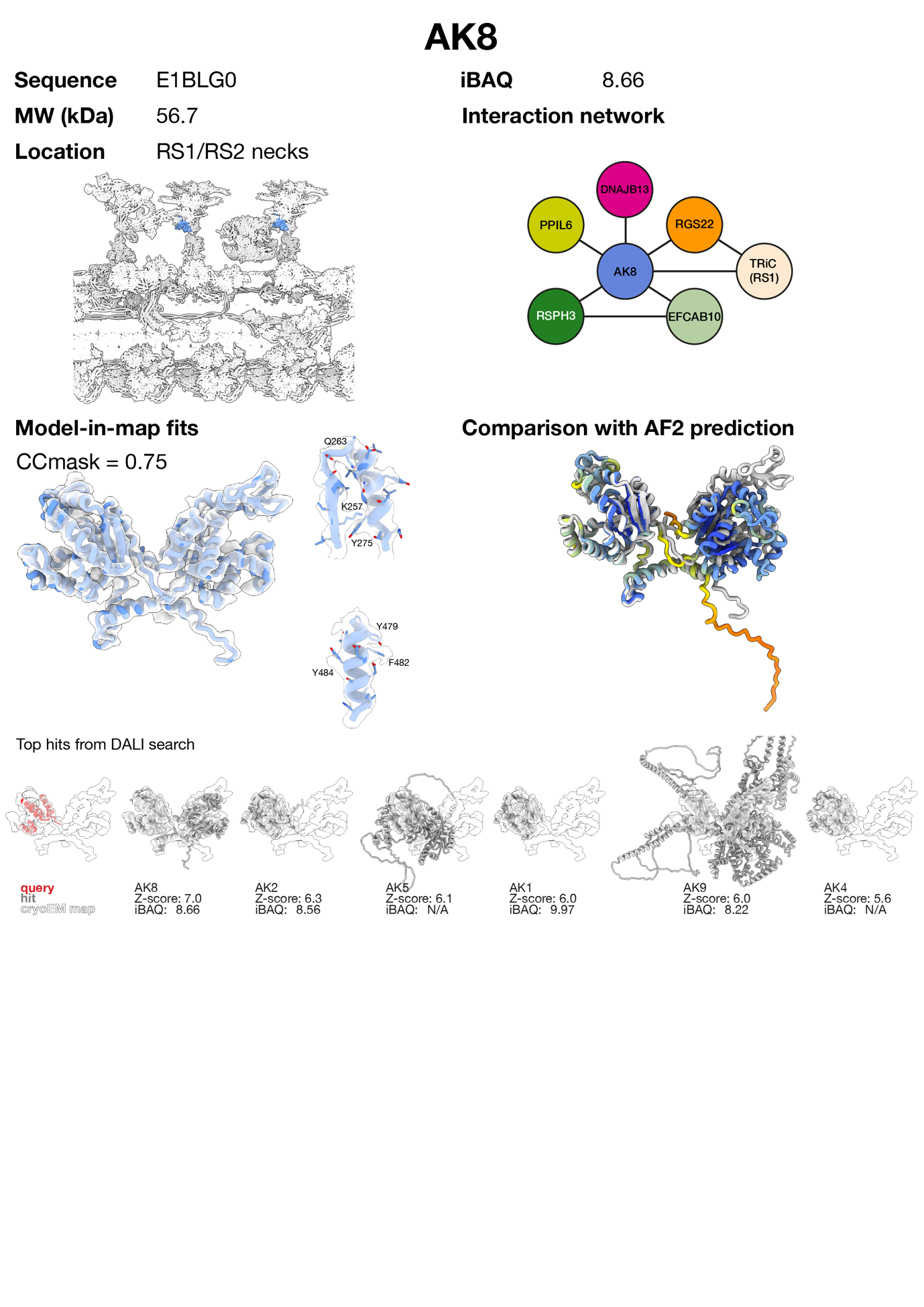
**

**Supplementary Figure 9 | Protein identification and assessment report for AK8.** AK8 was identified by manual tracing of helices followed by a DALI search against the AlphaFold database. Of the top hits, AK8 fit the density unambiguously (i.e. two adenylate kinase domains and an RIIa-like domain). Other hits were discarded because they did not match the density well and/or were not detected in the bovine sperm proteome. To confirm assignment using side chain density, a poly-Ala trace was used as input to findMySequence, which confidently identified AK8 (e-value 4.4e^-18^). Supporting evidence: (i) immunofluorescence localizes AK8 along the mouse sperm flagellum^6^, (ii) AK8 co-immunoprecipitates with RSPH3B, a known RS1/RS2 head protein, in mouse sperm flagella^7^, (iii) sperm from IQUB-knockout mice lacking RS1 show reduced levels of AK8 ^8^, (iv) AK8::GFP co-localizes with RSPH4A in ependymal cilia and co-immunoprecipitates with RSPH3B in HEK293T cells^9^, (v) AK8 levels are reduced in RSP3-knockout strains of *Tetrahymena*^5^, and (vi) AK8 cross-links to RSP3 in *Tetrahymena* axonemes^10^.


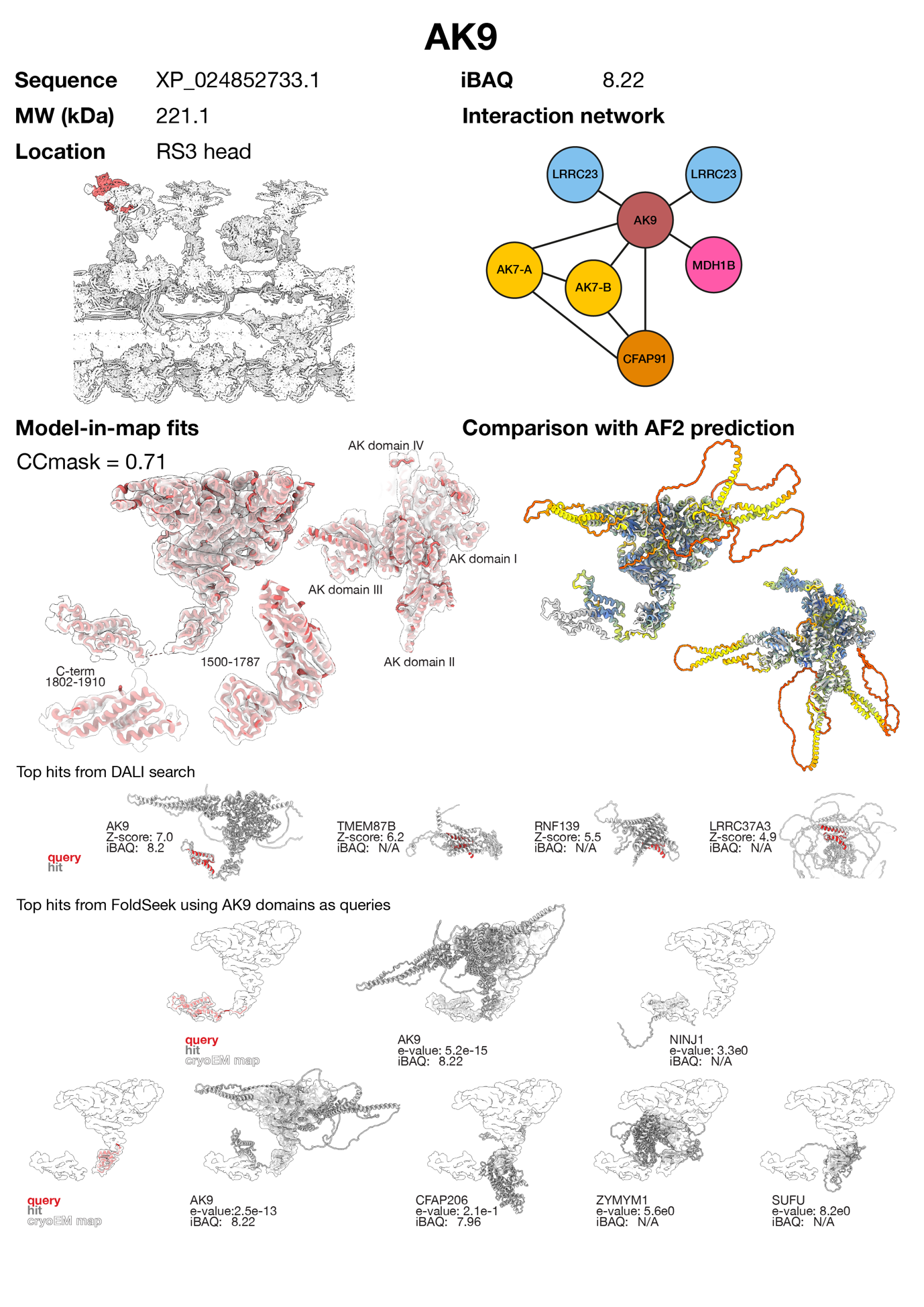


**Supplementary Figure 10 | Protein identification and assessment report for AK9.** The C-terminal domain of AK9 was identified by manual tracing of helices in Coot followed by a DALI search against the AlphaFold database. The remaining domains of AK9 matched neighbouring density. Other hits were discarded because they did not match the density well and were not detected in the bovine sperm proteome. To find alternatives with similar folds, the C-terminal domains of AK9 were separately used as inputs to FoldSeek to query the AlphaFold database. No hit matches the density as well as AK9. Supporting evidence: (i) immunofluorescence localizes AK9 along the axoneme in mouse^11^ and human sperm^12^, (ii) AK9 is absent in LRRC-deficient human sperm and in LRRC23^-/-^ mouse sperm ^7^, which also lack the RS3 head^13^, (iii) AK9 co-immunoprecipitates with the RS3 base protein CFAP251 in *Tetrahymena*^4^, (iv) AK9 levels are reduced when the RS3 protein CFAP91 is knocked out in *Tetrahymena*^5^, and (v) AK9::GFP localizes along the axoneme in *Xenopus* epidermal multi-ciliated cells^10^.


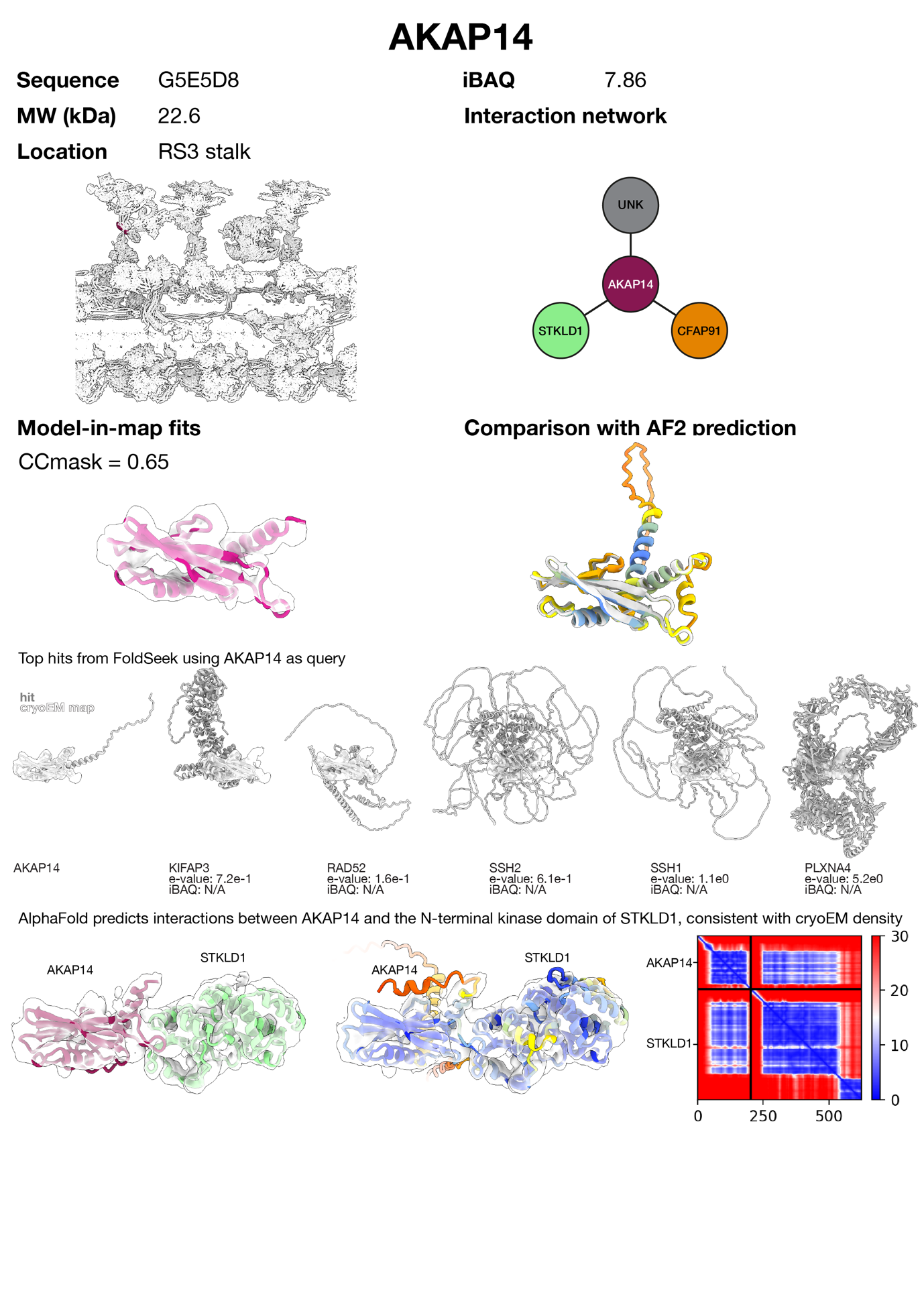


**Supplementary Figure 11 | Protein identification and assessment report for AKAP14.** The AlphaFold prediction for AKAP14 matches the density. Proteins with similar folds, identified using FoldSeek, do not match the density well and were absent from the bovine sperm proteome. AlphaFold-Multimer predicts interactions between AKAP14 and the N-terminal kinase domain of STKLD1, consistent with the cryo-EM density. Supporting evidence: AKAP14 is enriched in airway cilia^14^ and in other ciliated cell types^15^.


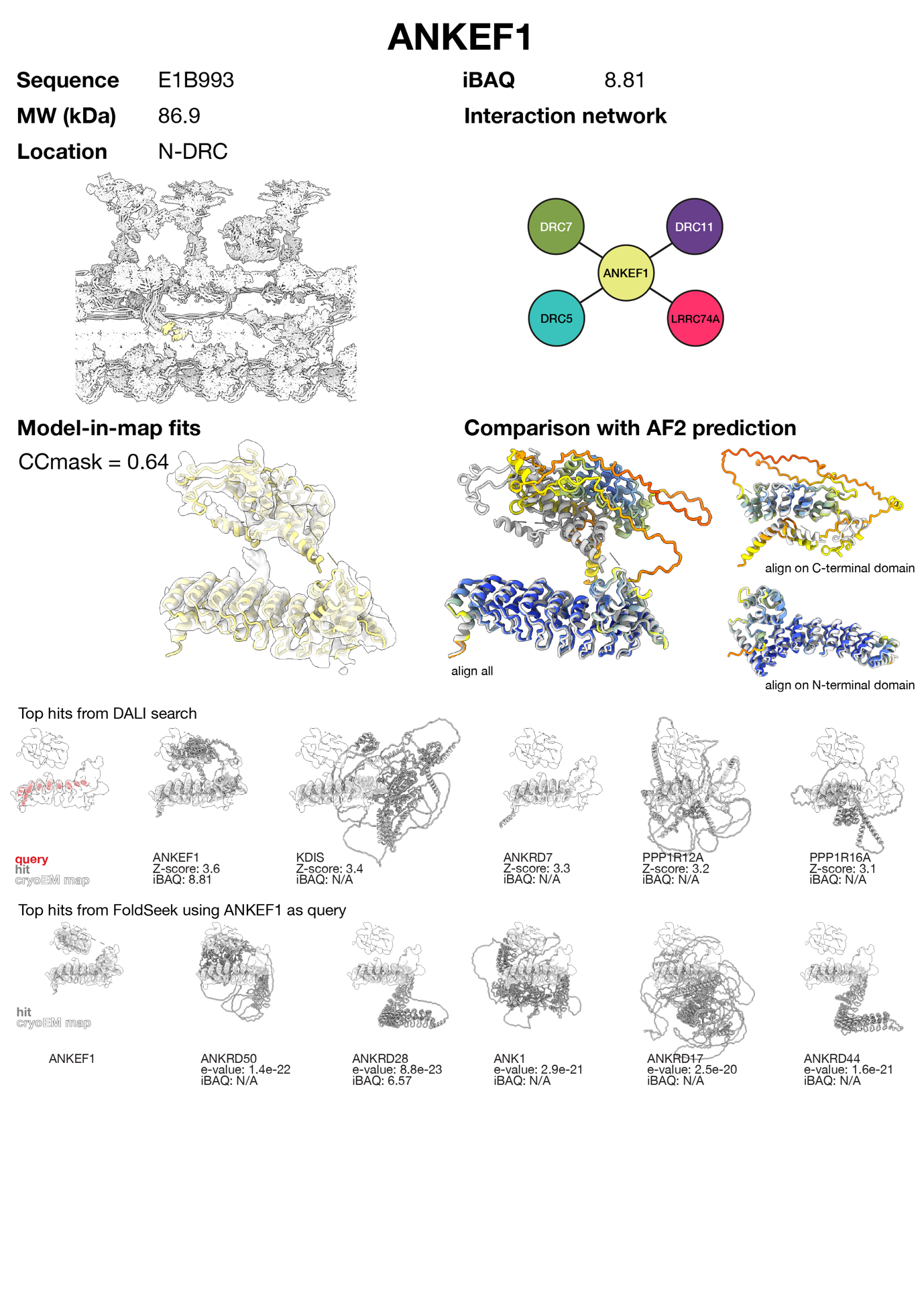


**Supplementary Figure 12 | Protein identification and assessment report for ANKEF1.** ANKEF1 was identified by manual tracing of helices in Coot followed by a DALI search against the human AlphaFold database. Other hits were discarded because they did not match the density well and were not detected in the bovine sperm proteome. Structurally similar proteins, identified using FoldSeek, do not match the density as well as ANKEF1. Supporting evidence: (i) ANKEF1 expression is enhanced in ciliated cells in zebrafish^16^ and human tissues^15^, and (ii) immunofluorescence localizes ANKEF1 to the axonemes of kinocilia in zebrafish hair cells^17^.


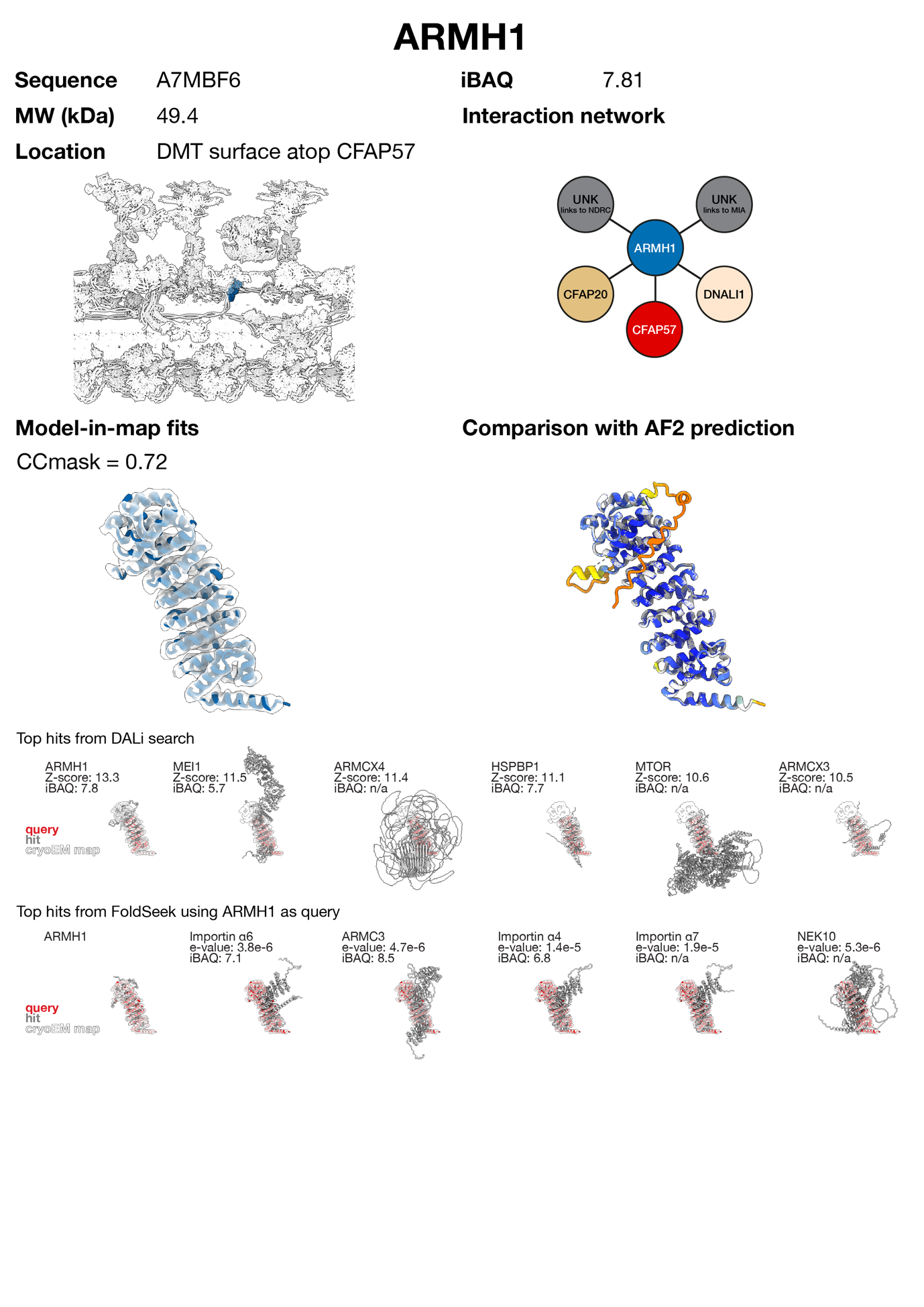


**Supplementary Figure 13 | Protein identification and assessment report for ARMH1.** ARMH1 was identified by manual tracing of helices followed by a DALI search against the AlphaFold database. Other hits were discarded because they did not match the density well and/or were less abundant or not detected in the bovine sperm proteome. Structurally similar proteins, identified using FoldSeek, do not match the density as well as ARMH1 and/or were less abundant or not detected in the bovine sperm proteome. Supporting evidence: ARMH1 expression is enriched in ciliated cells^15^.


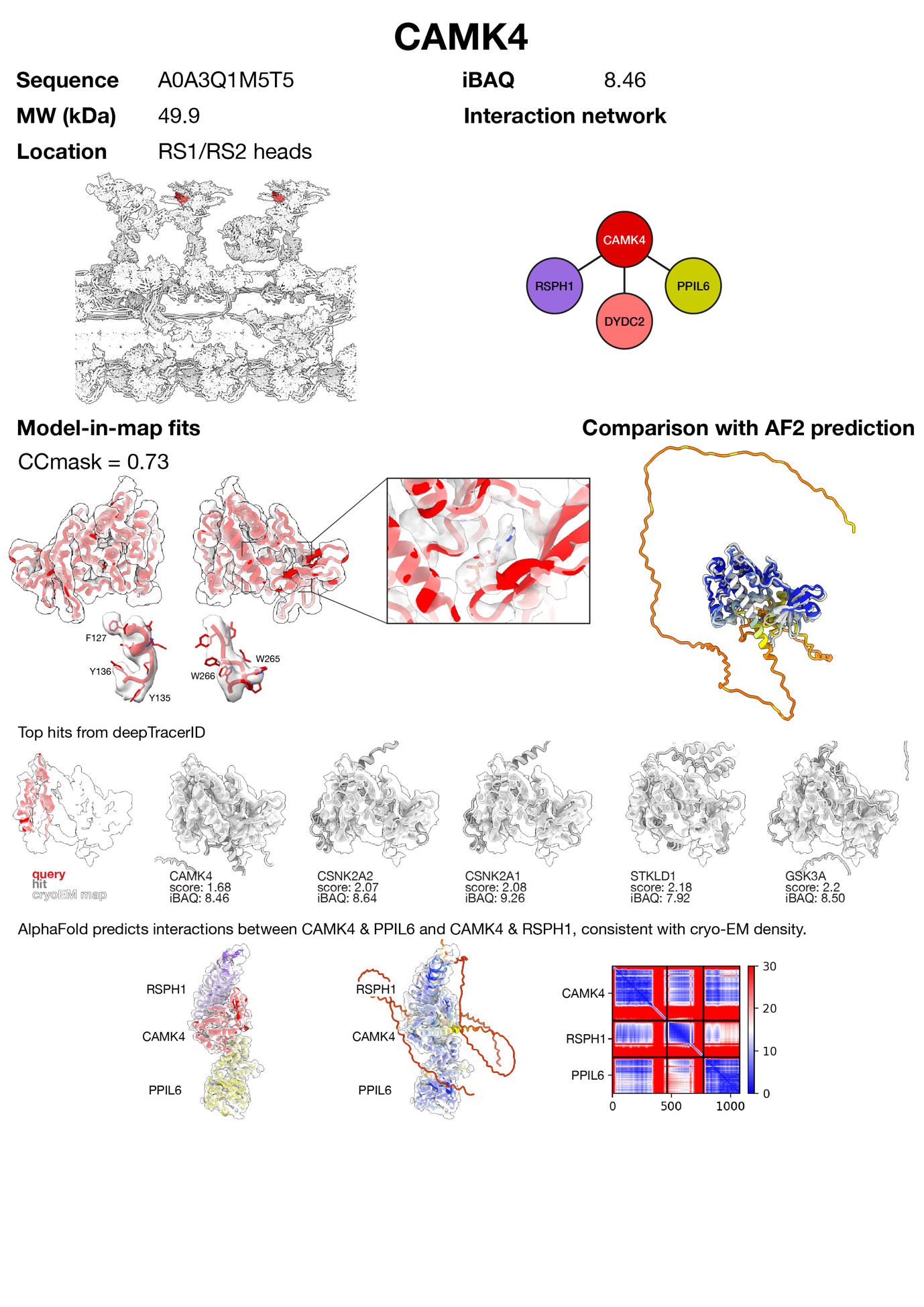


**Supplementary Figure 14 | Protein identification and assessment report for CAMK4.** CAMK4 was identified by manual tracing of helices followed by a deepTracerID search against an AlphaFold database of the bovine sperm proteome. To confirm assignment, a poly-Ala trace was used as input to findMySequence, which identified CAMK4 (e-value = 4.5e^-15^). AlphaFold-Multimer predicts interactions between CAMK4 & PPIL6 and CAMK4 & RSPH1, consistent with cryo-EM density. Supporting evidence: (i) immunofluorescence localizes CAMK4 to the flagellum in both human^18^ and chicken sperm^19^, and (ii) biochemical fractionation and immunofluorescence suggest CAMK4 is bound to the axoneme in tilapia sperm^20^. An alternative CAMK (CAMK2D) is less abundant in the sperm proteome (iBAQ = 5.31).


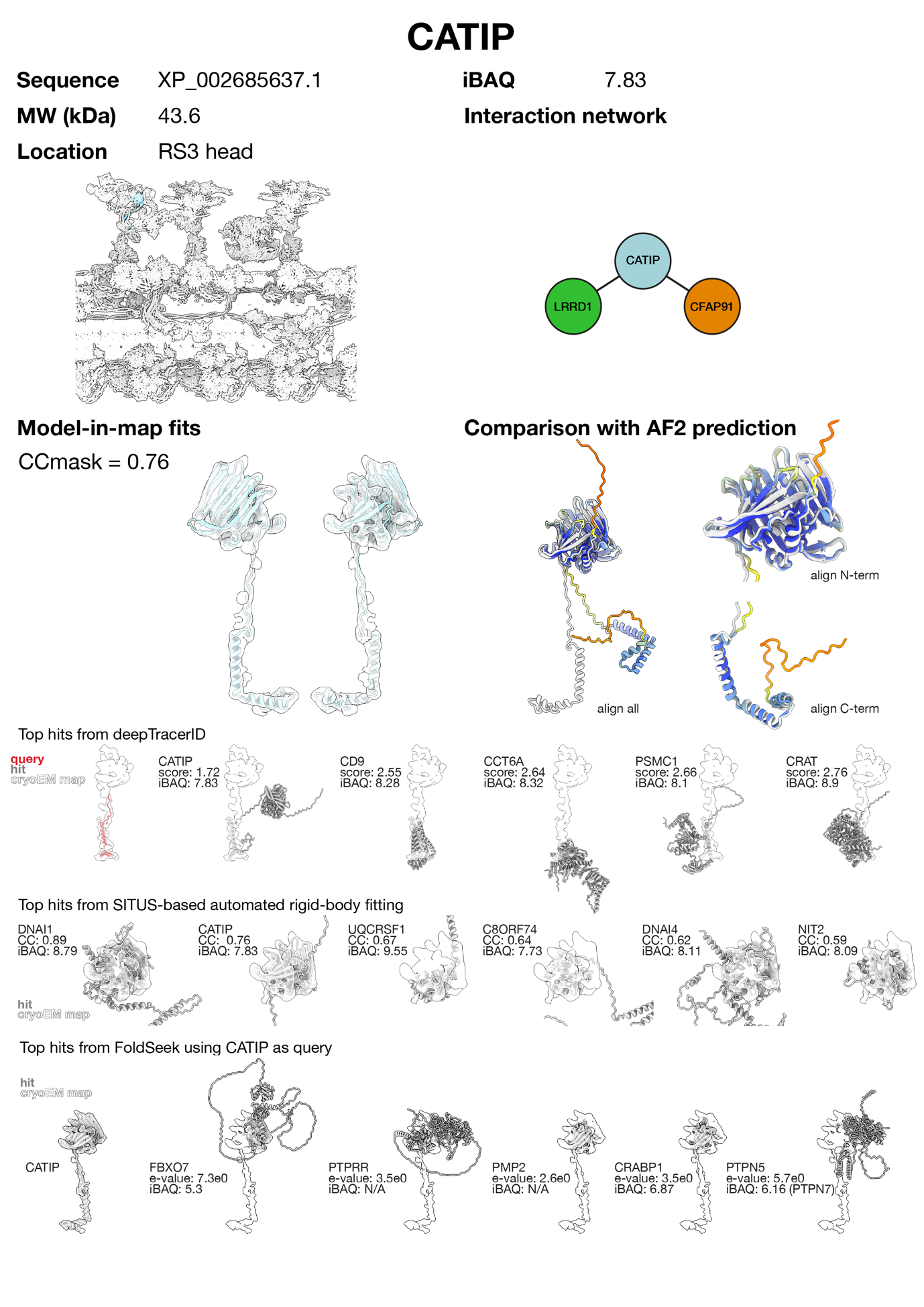


**Supplementary Figure 15 | Protein identification and assessment report for CATIP.** The C-terminal domain of CATIP was identified by manual tracing of helices followed by a deepTracerID search against an AlphaFold database constructed from the bovine sperm proteome. The N-terminal domain of CATIP matched neighbouring density. Other hits were discarded because they did not match the density well. The N-terminal domain of CATIP was independently identified using SITUS to automatically rigid-body fit a library of AlphaFold predictions from the bovine sperm proteome. Other hits were discarded as they did not fit the density well. Structurally similar proteins, identified using FoldSeek, do not match the density as well as CATIP and/or were not detected in the bovine sperm proteome. Supporting evidence: immunofluorescence and immuno-histochemistry localize CATIP to ciliated cells in zebrafish and human tissues^21^.


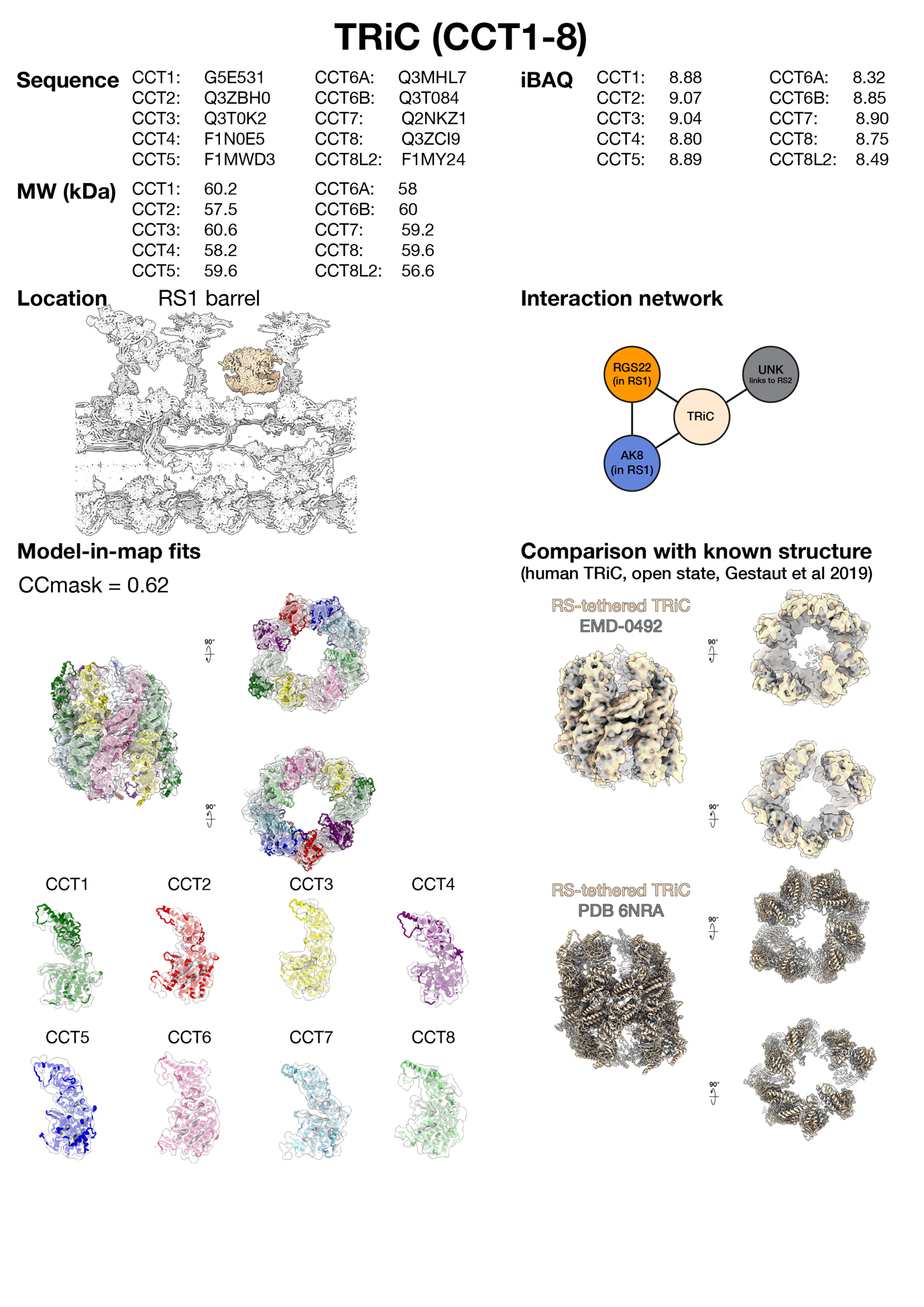


**Supplementary Figure 16 | Protein identification and assessment report for TRiC (CCT1-8).** The overall size, shape, and arrangement (i.e. two stacked rings of eight subunits each) of the barrel-shaped complex matches the distinct structure of the TRiC chaperone complex. The structure of individual CCT subunits matches the cryo-EM density. Supporting evidence: Immunofluorescence localizes CCT subunits to mouse sperm flagella^22,23^.


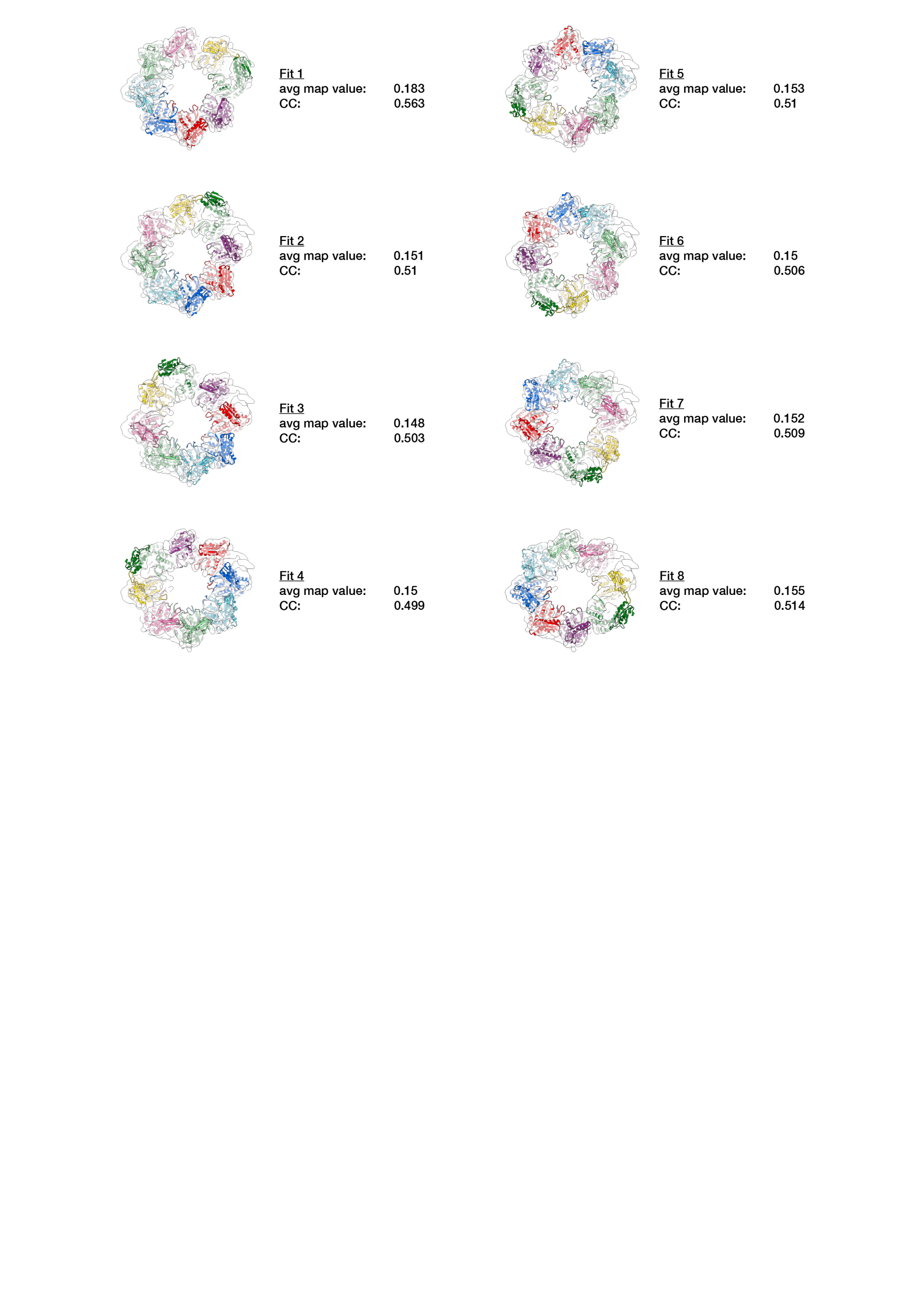


**Supplementary Figure 17 | Assigning subunit order in RS-tethered TRiC.** CCT subunit order was assigned based on distinct features of open-state TRiC that are conserved from yeast to mammals^24^; specifically, that CCT1 is the most outwardly-tilted and that the largest gap in the ring occurs between CCT1 and CCT4. To confirm subunit assignment, we took a model of open-state TRiC (PDB 7YLV ^25^), then calculated average map values and cross-correlation scores for eight possible rotations, which showed that our assignment was the best-fitting solution.


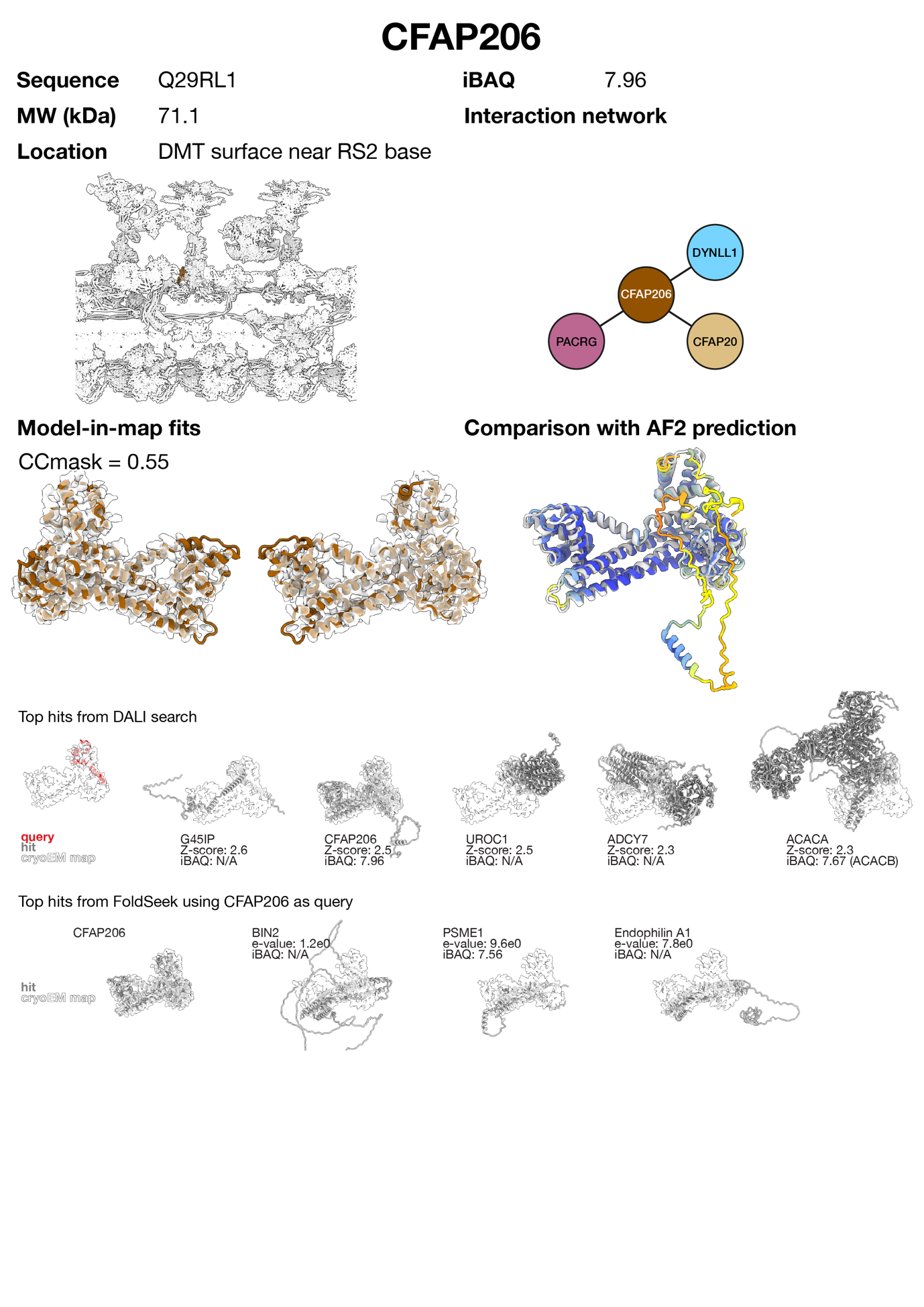


**Supplementary Figure 18 | Protein identification and assessment report for CFAP206.** CFAP206 was identified by automated tracing using ModelAngelo followed by a DALI search against the human AlphaFold database. Other hits were discarded because they did not match the density. Structurally similar proteins, identified using FoldSeek, do not fit the density as well as CFAP206. Supporting evidence: (i) immunofluorescence localizes CFAP206 along axonemes of respiratory epithelia^26^, (ii) CFAP206::GFP localizes along axonemes of *Xenopus* epidermal multiciliated cells^26^, (iii) *Tetrahymena* knockouts of CFAP206 lack RS2 and IDA*c*^27^.


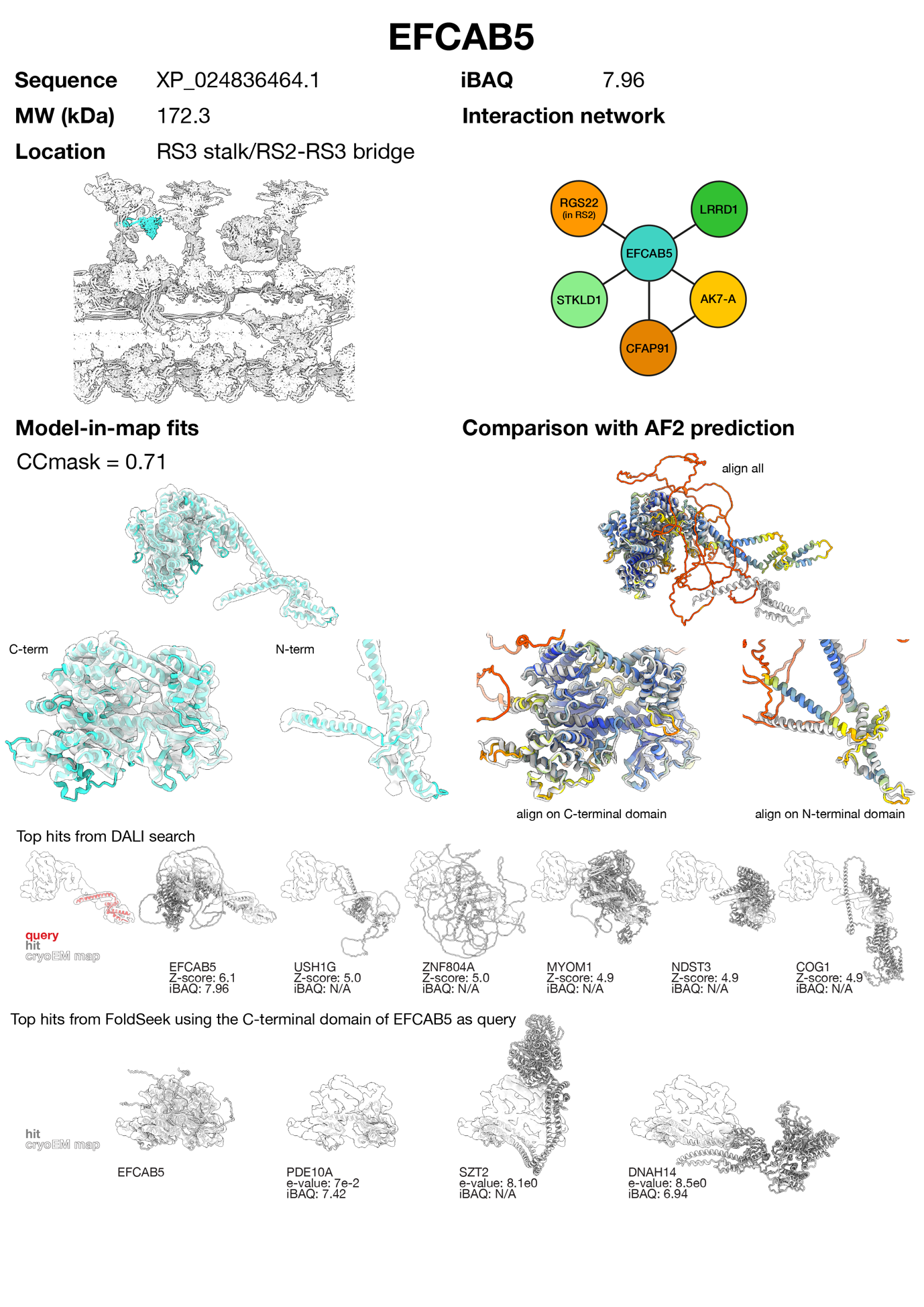


**Supplementary Figure 19 | Protein identification and assessment report for EFCAB5.** The N-terminus of EFCAB5 was identified by a DALI search against the AlphaFold database. The rest of EFCAB5 matched adjacent density. Other hits were discarded because they did not fit the density well and were not detected in the bovine sperm proteome. To find alternative possibilities with similar folds, the C-terminal globular domain of EFCAB5 was used to query the AlphaFold database using FoldSeek. None of the other top hits fit the density.


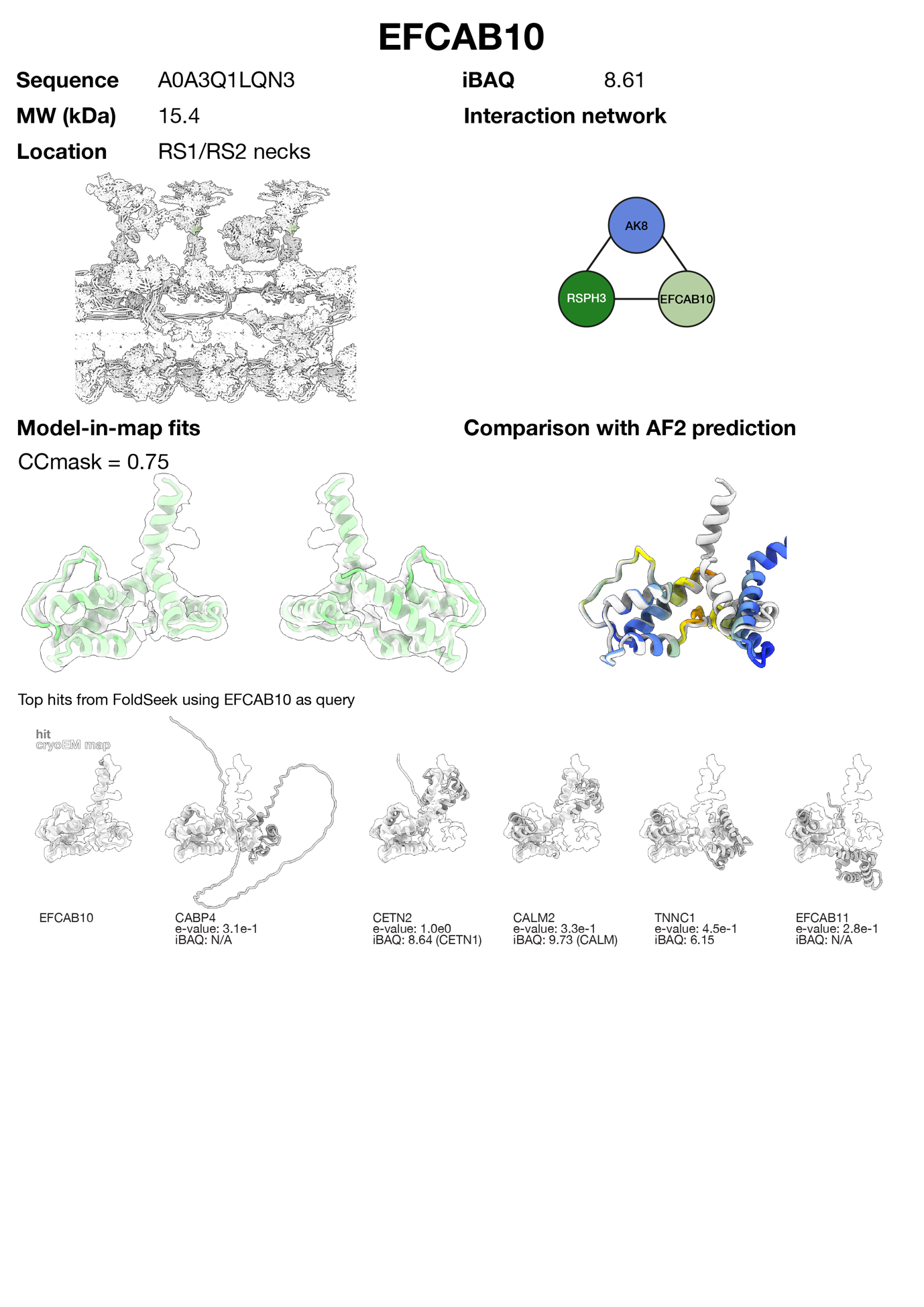


**Supplementary Figure 20 | Protein identification and assessment report for EFCAB10.** EFCAB10 is the ortholog of the recently identified *C. reinhardtii* protein RSP26 that binds to the same location of RS1/2 ^28^. The AlphaFold prediction of EFCAB10 fits the density well. Structurally similar proteins, identified using FoldSeek, do not fit the density as well as EFCAB10. Supporting evidence: EFCAB10 expression is enriched in ciliated cells^15^.


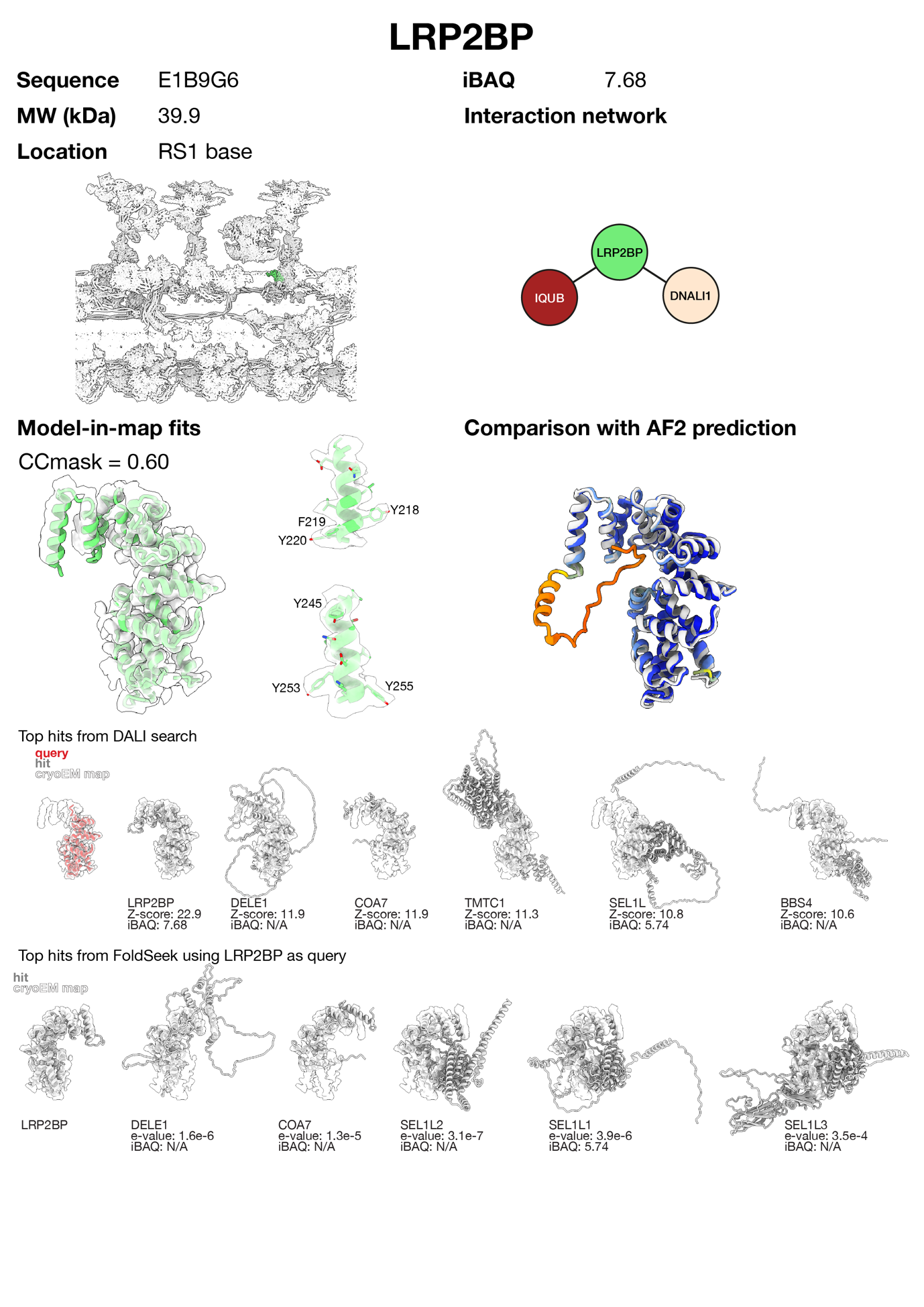


**Supplementary Figure 21 | Protein identification and assessment report for LRP2BP.** LRP2BP was identified by a DALI search against the AlphaFold database using a backbone traced automatically with ModelAngelo and adjusted in Coot. Other hits were discarded because they did not fit the density as well as LRP2BP. Structurally similar proteins, identified using FoldSeek, do not fit density as well as LRP2BP. To confirm assignment using side chain density, a poly-Ala trace was used as input to findMySequence, which confidently identified LRP2BP (e-value 1.9e^-29^).


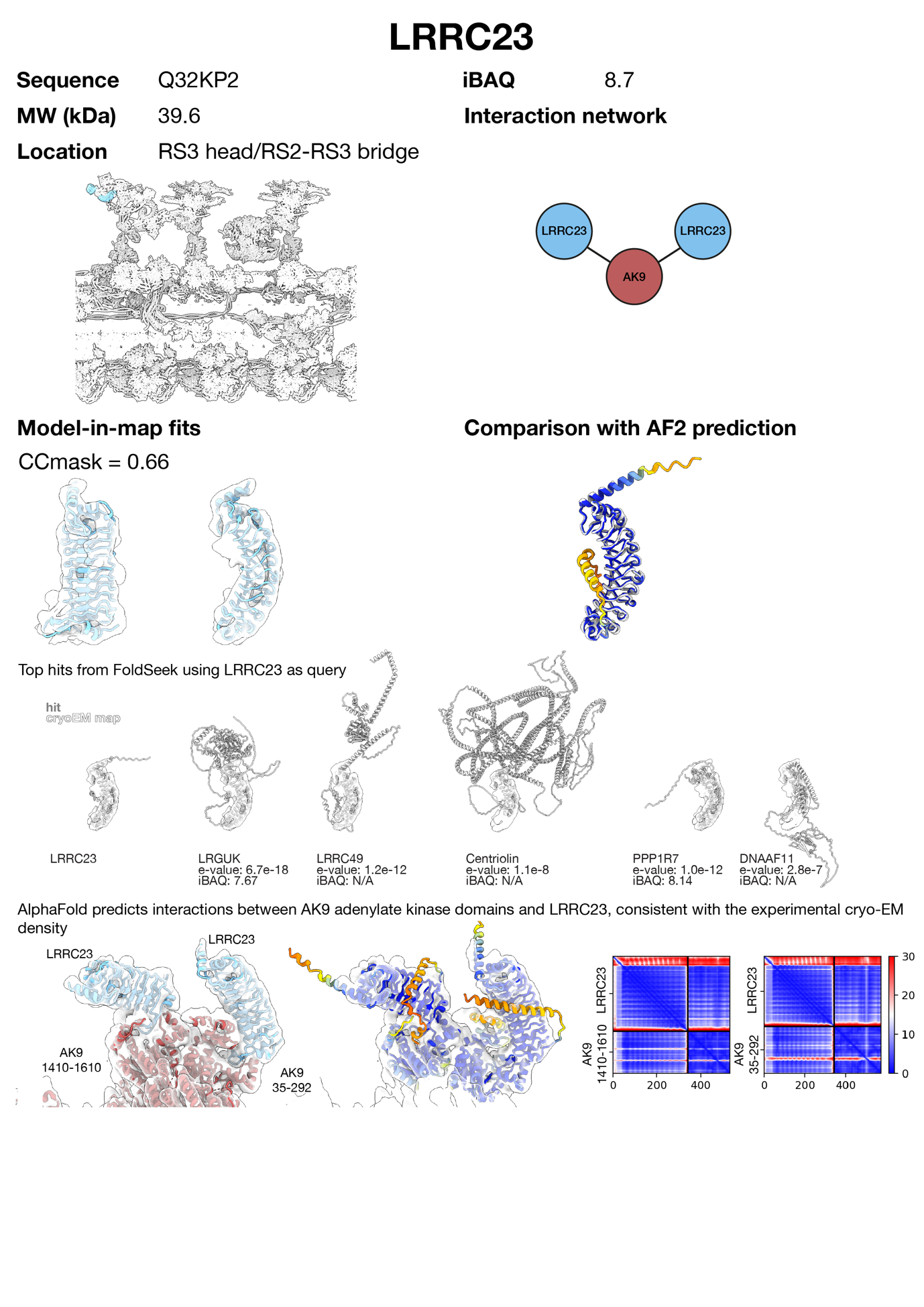


**Supplementary Figure 22 | Protein identification and assessment report for LRRC23.** The AlphaFold prediction for LRRC23 matches the cryo-EM density well. To find alternatives with similar folds, LRRC23 was input as query to FoldSeek searching the AlphaFold database. Top hits were discarded because they did not match the density as well as LRRC23 and/or were less abundant/not detected in the bovine sperm proteome. AlphaFold-Multimer predicts interactions between AK9 adenylate kinase domains and LRRC23, consistent with the experimental cryo-EM density. Supporting evidence: (i) immunogold labelling localizes LRRC23 to the radial spokes in mouse sperm flagella^29^, and (ii) LRRC23 truncation leads to loss of the RS3 head in mouse sperm^13^.


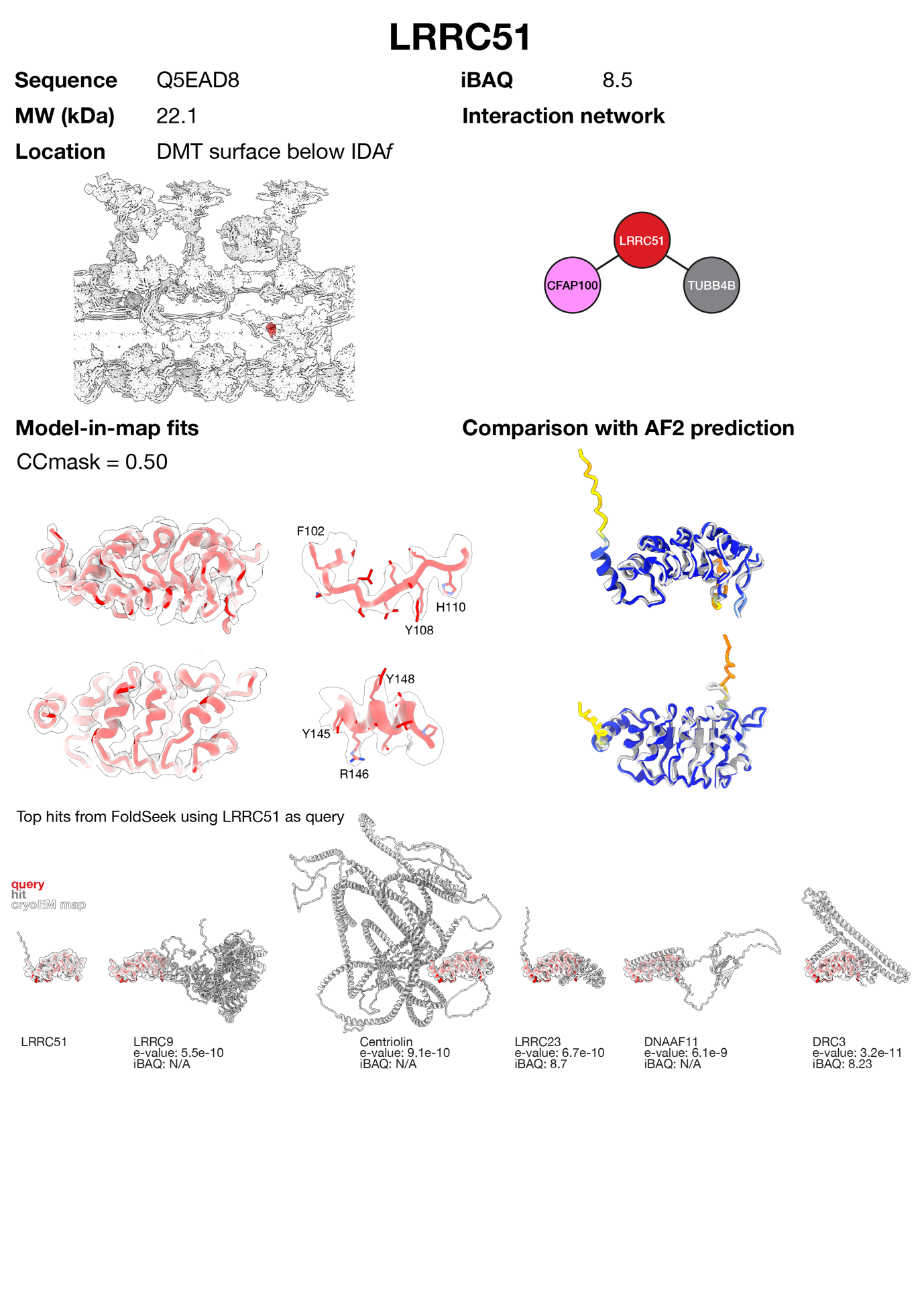


**Supplementary Figure 23 | Protein identification and assessment report for LRRC51.** The AlphaFold prediction for LRRC51 fits the density unambiguously. To find alternative possibilities with similar folds, LRRC51 was input as a query to FoldSeek searching the AlphaFold database. Other hits were discarded because they did not match the density well and/or were less abundant/not detected in the bovine sperm proteome. To confirm assignment using side chain density, a poly-Ala trace was used as input to findMySequence, which confidently identified LRRC51 (e-value 3e^-17^). Supporting evidence: LRRC51 expression is enhanced in ciliated cells^15^.


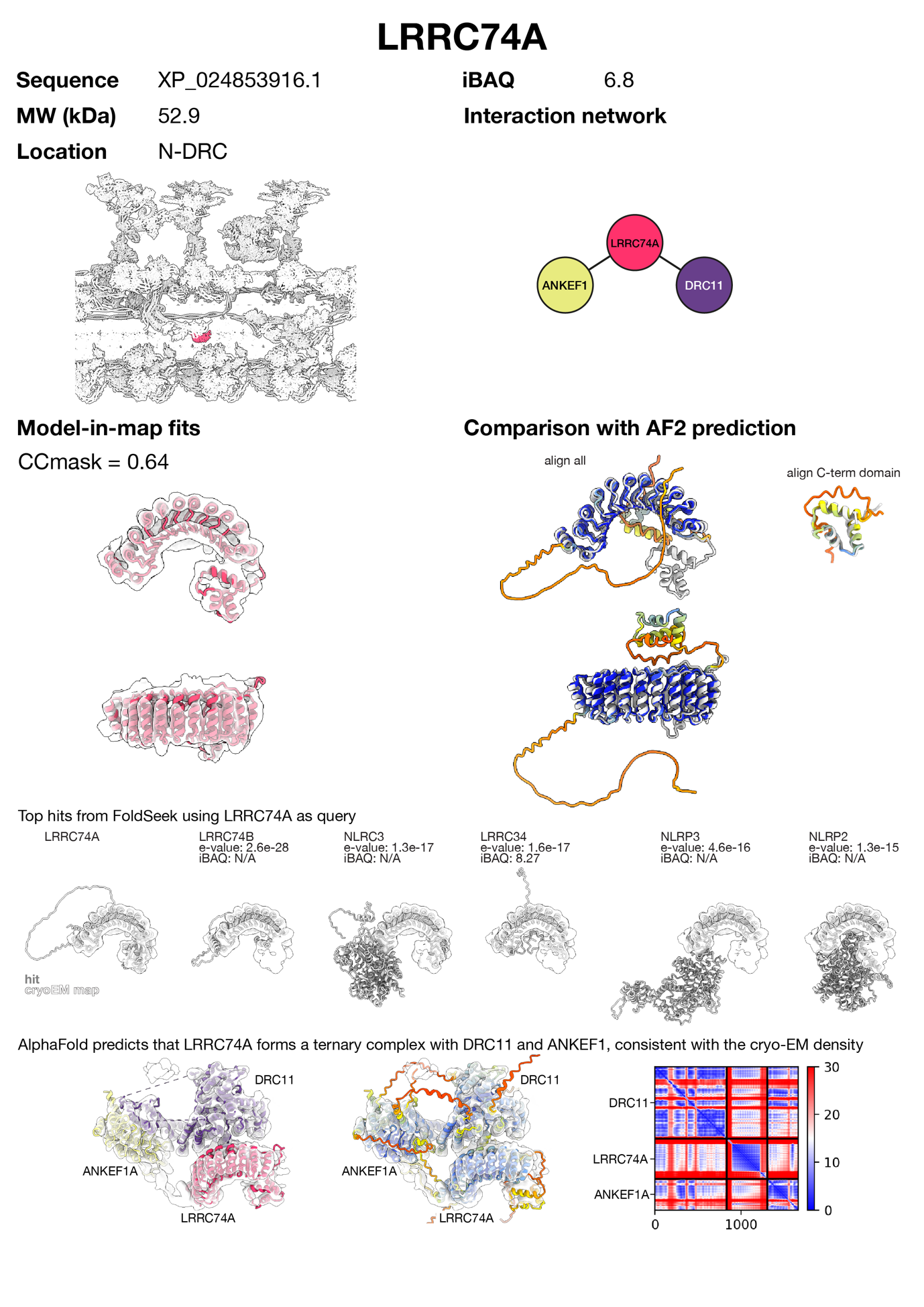


**Supplementary Figure 24 | Protein identification and assessment report for LRRC74A.** AlphaFold prediction for LRRC74A matches the density. FoldSeek-identified alternatives did not match the density as well as LRRC74A (in particular the C-terminal globular domain) and/or were less abundant or not detected in the bovine sperm proteome. AlphaFold-Multimer predicts that LRRC74A forms a ternary complex with DRC11 and ANKEF1, consistent with the cryo-EM density.


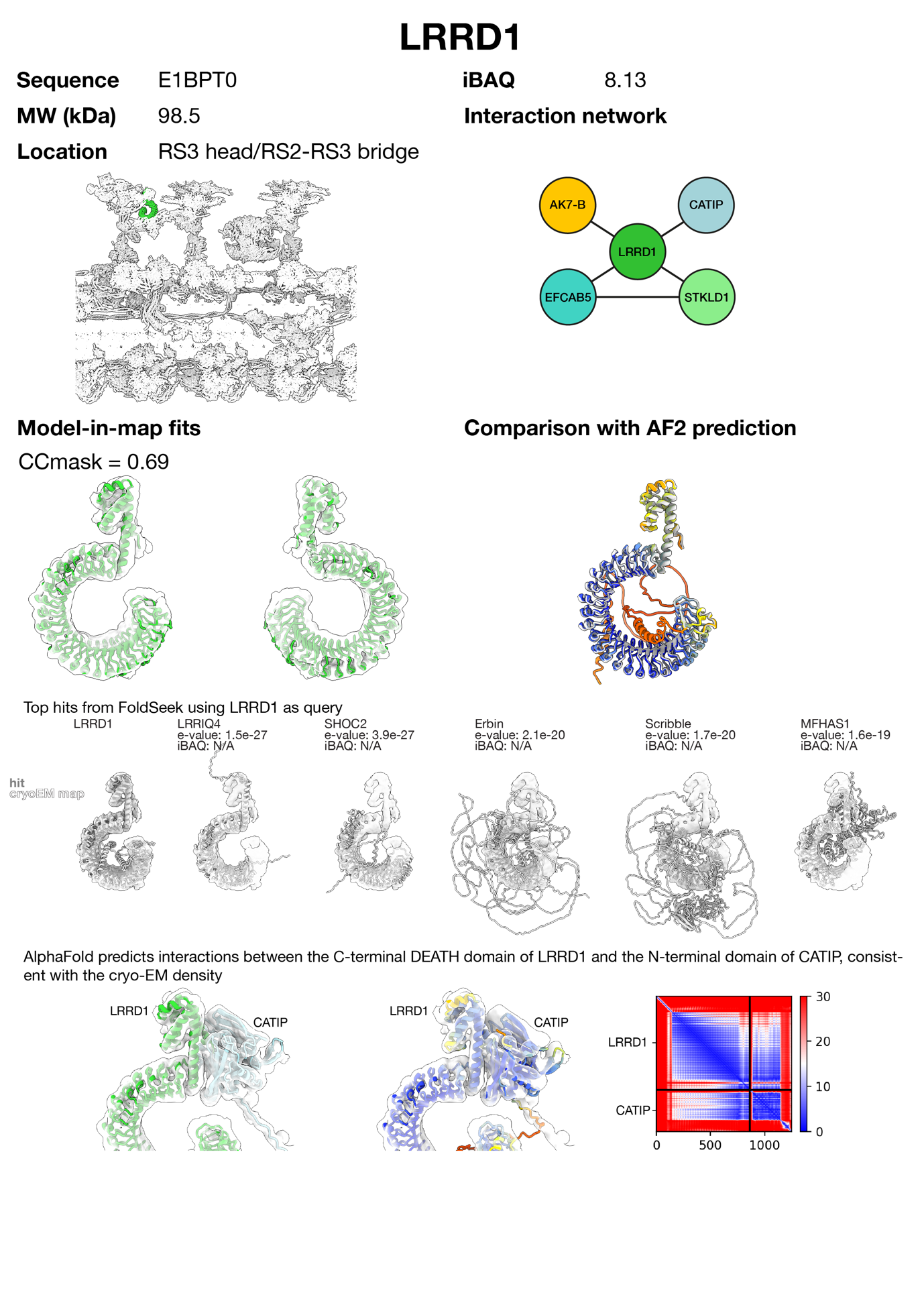


**Supplementary Figure 25 | Protein identification and assessment report for LRRD1.** The AlphaFold prediction for LRRD1 matches the density unambiguously. To find alternative possibilities with similar folds, LRRD1 was input as query to FoldSeek searching the AlphaFold database. Top hits were discarded because they did not match the density as well as LRRD1 and/or were not detected in the bovine sperm proteome. AlphaFold predicts interactions between the C-terminal DEATH domain of LRRD1 and the N-terminal domain of CATIP, consistent with the cryo-EM density.


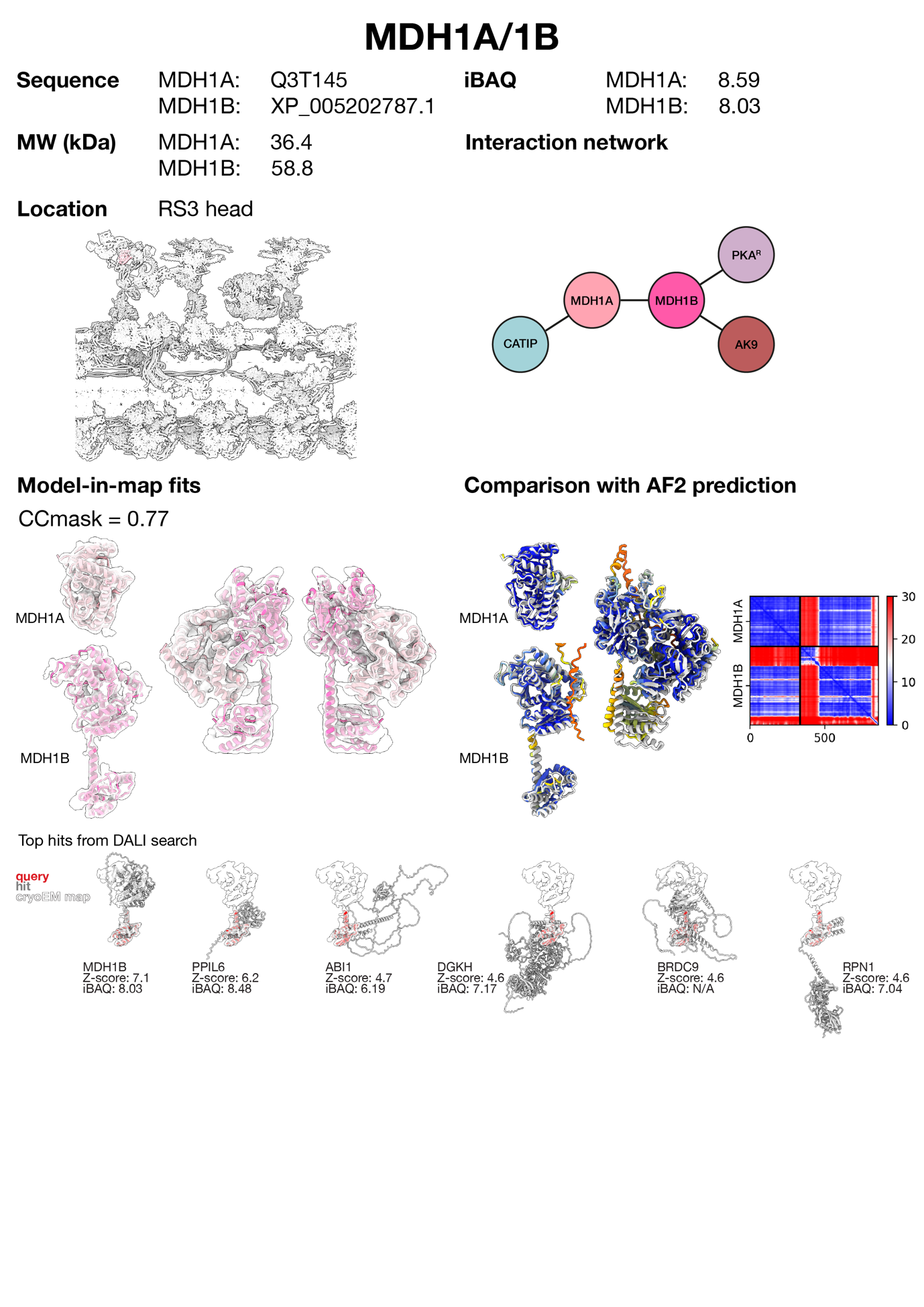


**Supplementary Figure 26 | Protein identification and assessment report for MDH1A/1B.** MDH1B was assigned by manual tracing of helices followed by a DALI search against the AlphaFold database, which identified its N-terminal domain. The C-terminal dehydrogenase domain matched neighbouring density. Other hits were discarded because they did not match the density well and/or were less abundant/not detected in the bovine sperm proteome. Malate dehydrogenases are known to exist as dimers. The density beside MDH1B matches the dehydrogenase domain unambiguously, and the lack of a glutaredoxin-like domain suggests it is MDH1A and not a second copy of MDH1B. AlphaFold predicts interactions between MDH1A and MDH1B consistent with the experimental structure.


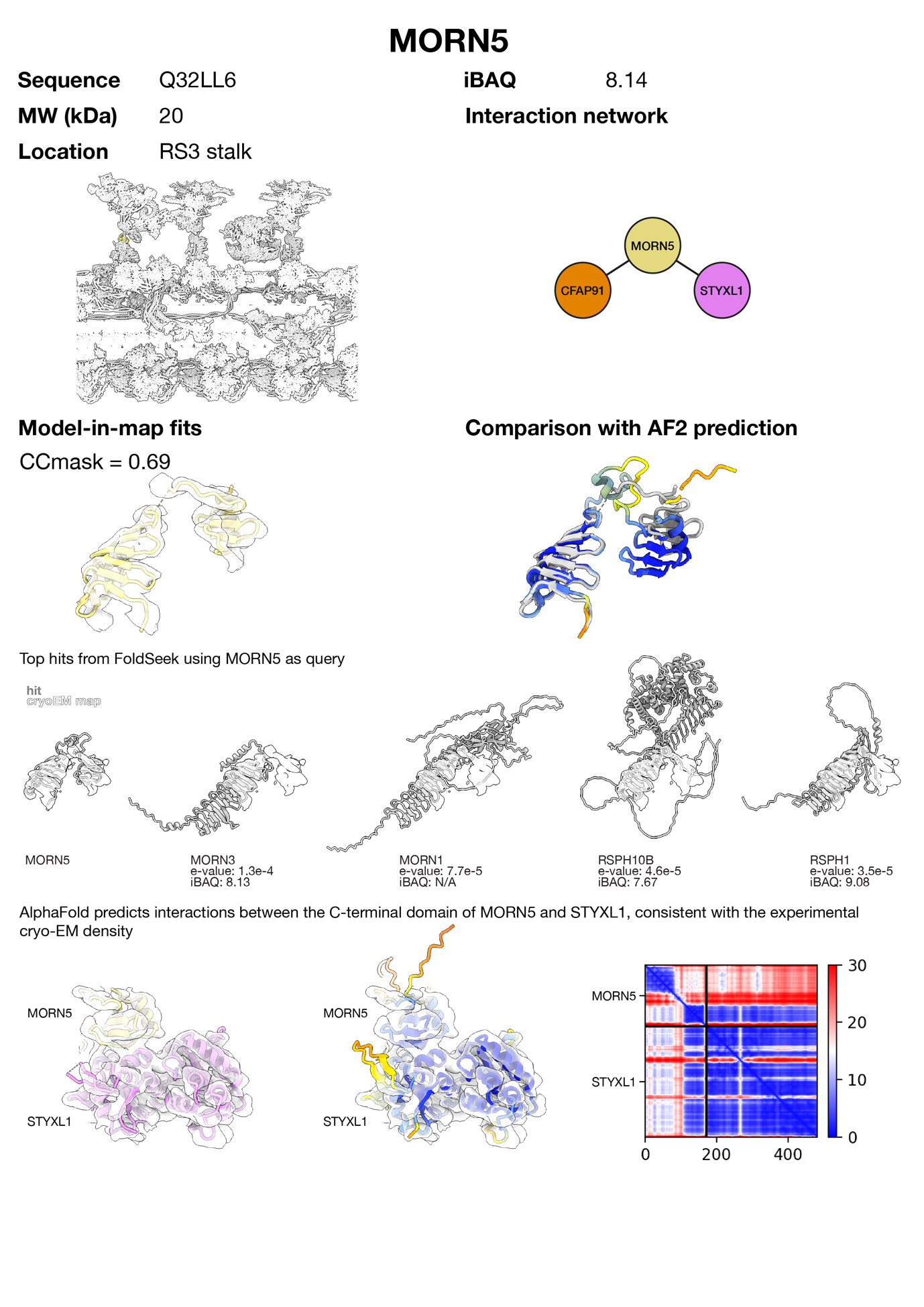


**Supplementary Figure 27 | Protein identification and assessment report for MORN5.** The AlphaFold prediction for MORN5 matches the density. No structurally similar protein, identified using FoldSeek, matches the density as well as MORN5. Additional domains present in the other top hits cannot be accounted for by neighbouring densities in RS3. AlphaFold predicts interactions between the C-terminal domain of MORN5 and STYXL1, consistent with the experimental cryo-EM density.


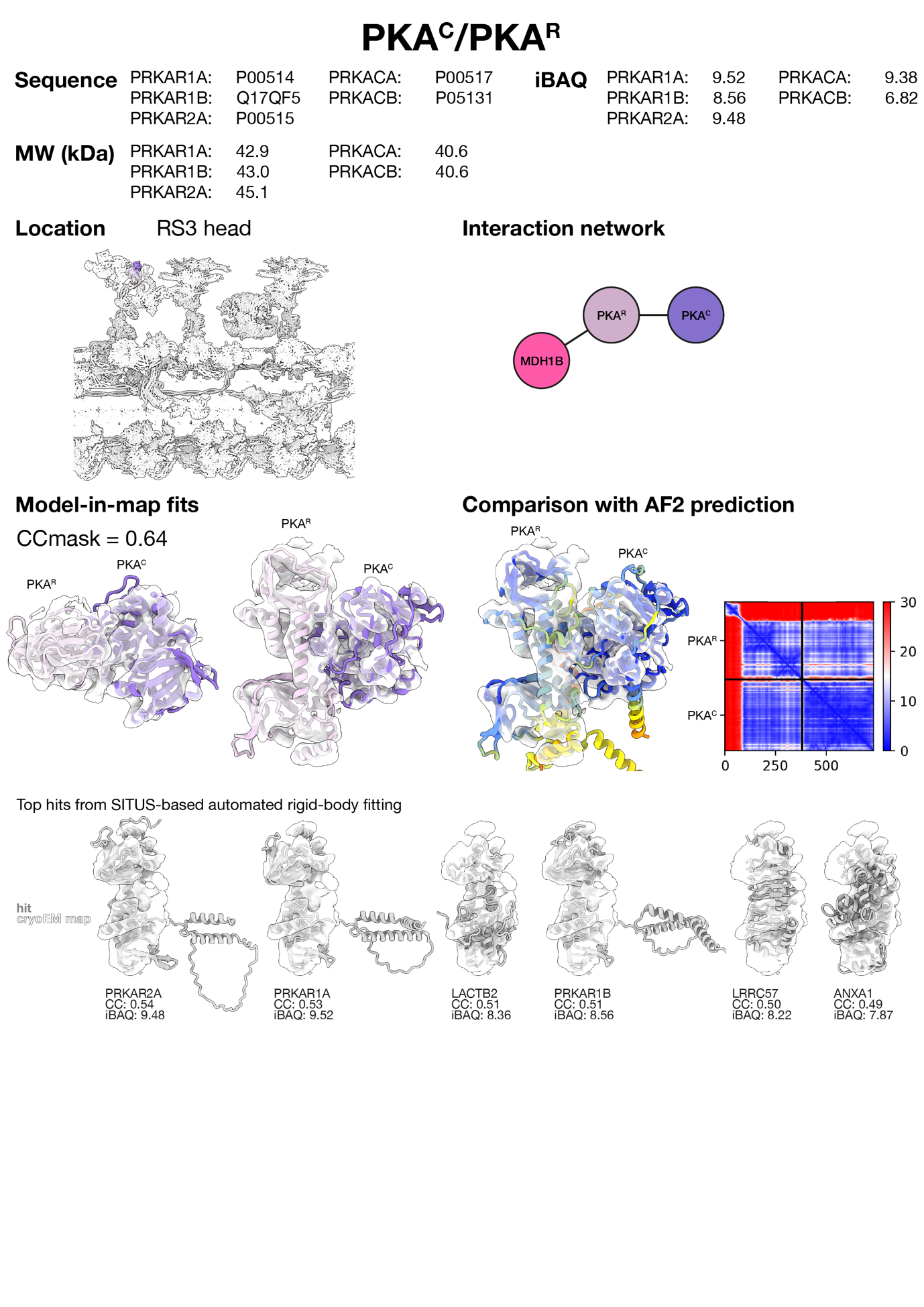


**Supplementary Figure 28 | Protein identification and assessment report for PKA^C^/PKA^R^.** PKA^R^ was identified by SITUS-based automated rigid-body fitting of an AlphaFold library of the bovine sperm proteome. Hits other than PKA regulatory subunits were discarded because they did not match the density as well. Note that we cannot determine the specific PKA regulatory subunit at the resolution of our maps. Density bound to PKA^R^ matches the AlphaFold prediction for PKA^C^, and the density for the complex matches the AlphaFold prediction for the PKA^R^/PKA^C^ dimer. Supporting evidence: (i) super-resolution microscopy localizes PKA^R^ and PKA^C^ to the axoneme in mouse sperm^30,31^, (ii) immunofluorescence studies^32,33^ show flagellar localization for PKA in sperm, and (iii) PKA co-purifies with axonemes from respiratory cilia^14^. Similar biochemical evidence supporting PKA being axoneme-associated is reviewed in ^34^.


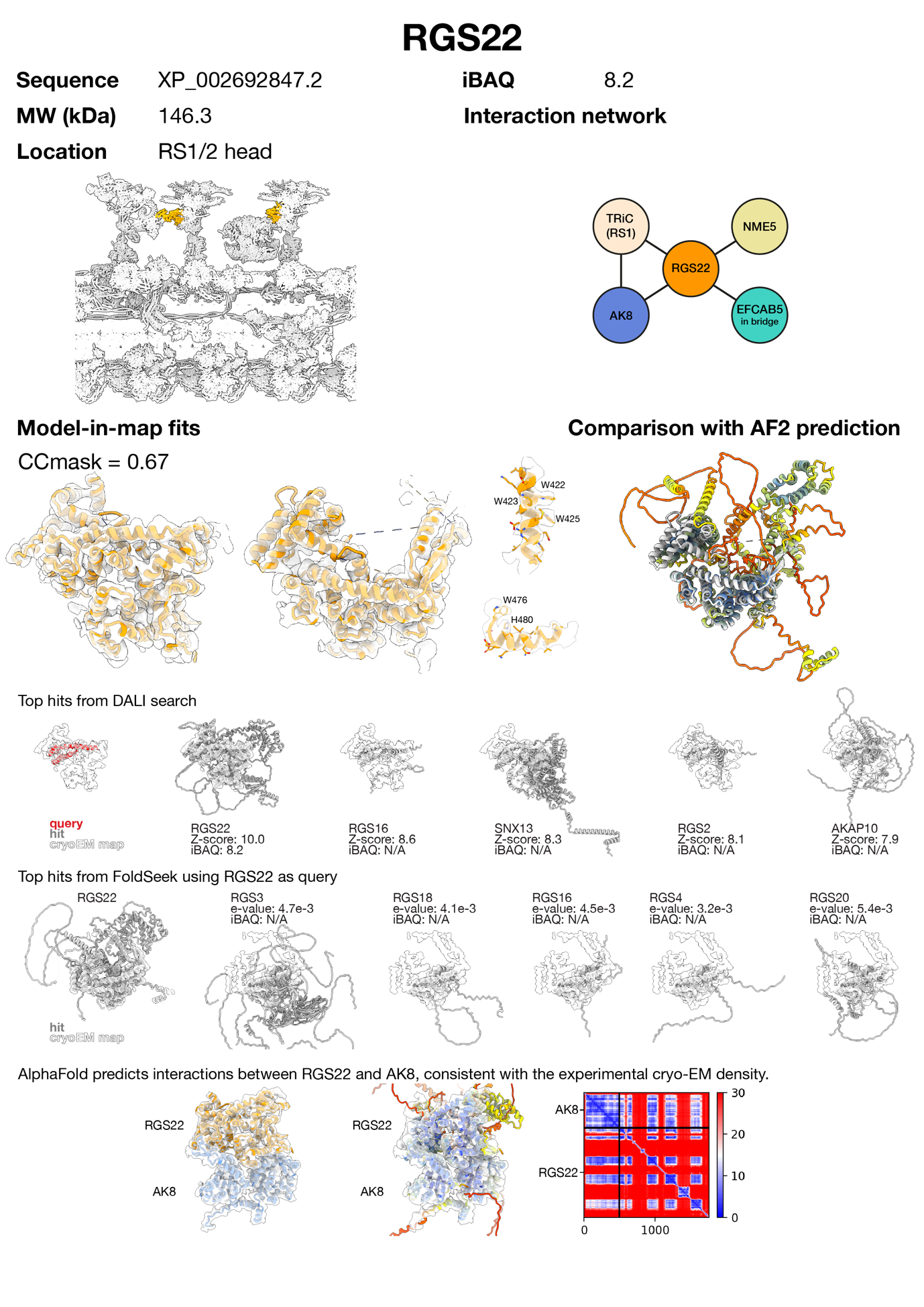


**Supplementary Figure 29 | Protein identification and assessment report for RGS22.** RGS22 was identified by manual tracing of helices followed by a DALI search against the AlphaFold database. Structurally similar proteins, identified using FoldSeek, do not match the density well and were absent from the bovine sperm proteome. AlphaFold-Multimer predicts interactions between RGS22 and AK8, consistent with the experimental cryo-EM density.


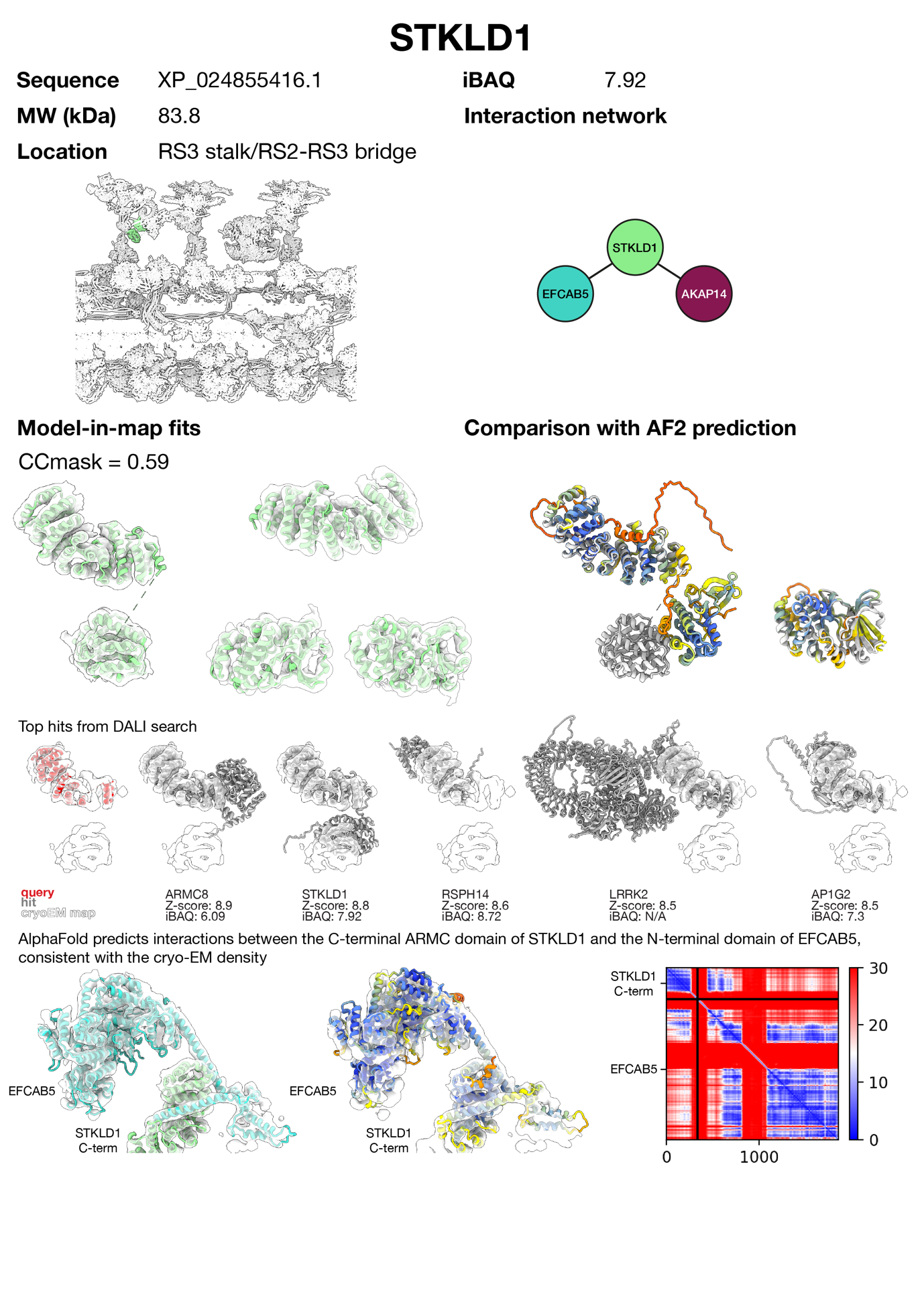


**Supplementary Figure 30 | Protein identification and assessment report for STKLD1.** The C-terminal ARM domain of STKLD1 was identified by manual tracing of helices followed by a DALI search against the AlphaFold database. The N-terminal kinase domain of STKLD1 matched the neighbouring density. Other hits were discarded because they did not match the density well and/or were less abundant/not detected in the bovine sperm proteome. AlphaFold-Multimer predicts interactions between the C-terminal ARMC domain of STKLD1 and the N-terminal domain of EFCAB5, consistent with the cryo-EM density.


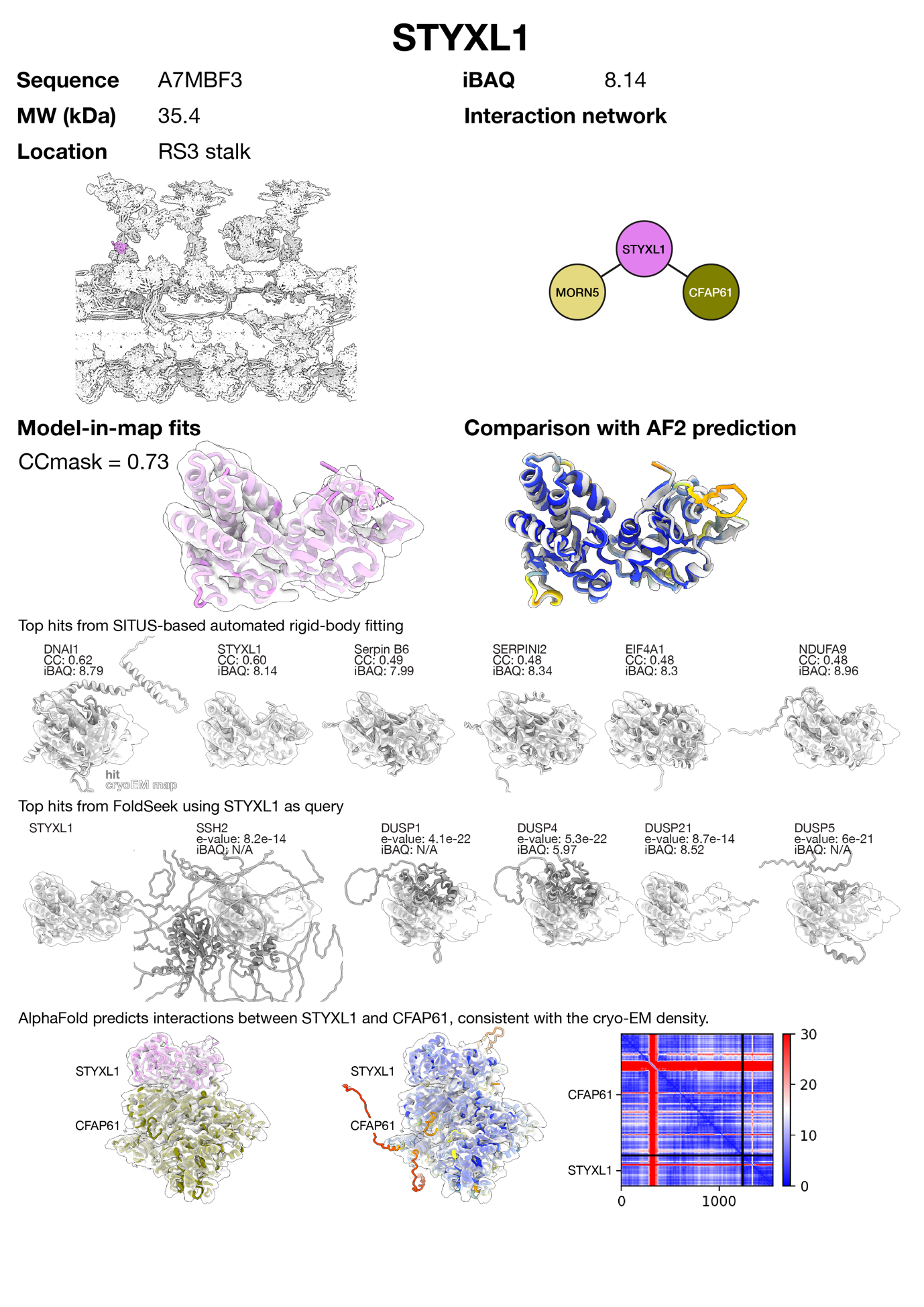


**Supplementary Figure 31 | Protein identification and assessment report for STKLD1.** STYXL1 was identified by SITUS-based automated rigid-body fitting of an AlphaFold library of the bovine sperm proteome. Other hits were discarded because they did not match the density well. Proteins with similar folds to STYXL1, identified using FoldSeek, do not match the density as well as STYXL1 and/or were not detected in the bovine sperm proteome. AlphaFold-Multimer predicts interactions between STYXL1 and CFAP61, consistent with the cryo-EM density.


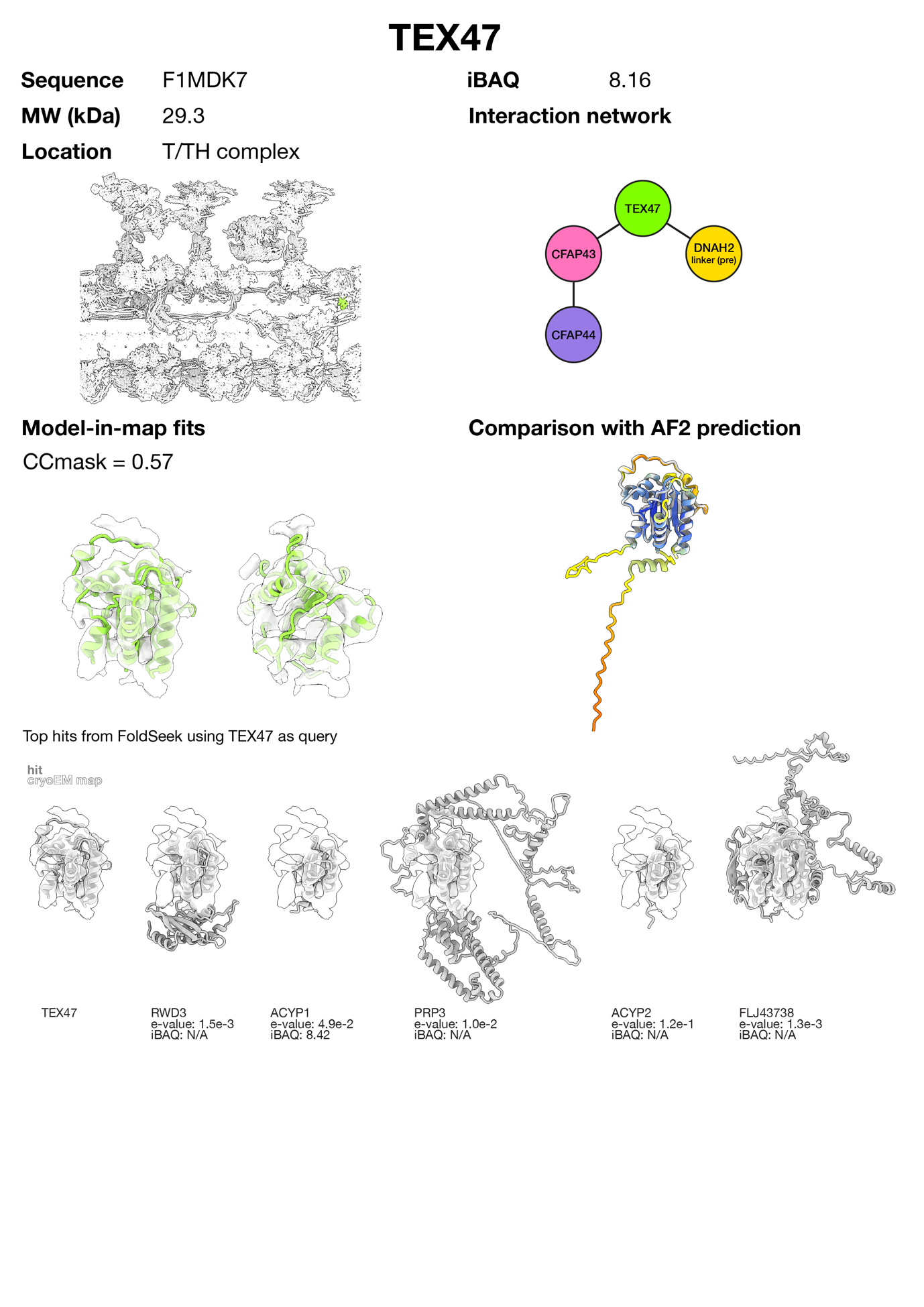


**Supplementary Figure 32 | Protein identification and assessment report for TEX47.** TEX47 is the ortholog of *C. reinhardtii* protein MOT7 that binds to the same location of the T/TH complex^35^. The AlphaFold prediction for TEX47 is a good match to the cryo-EM density. To find alternative possibilities with similar folds, TEX47 was input as query to FoldSeek searching the whole human AlphaFold database. Other hits were discarded because they did not match the density as well as TEX47 and/or were not detected in the bovine sperm proteome. Supporting evidence: the related *Leishmania* LAX28 protein is associated with the T/TH protein CFAP44^36^.


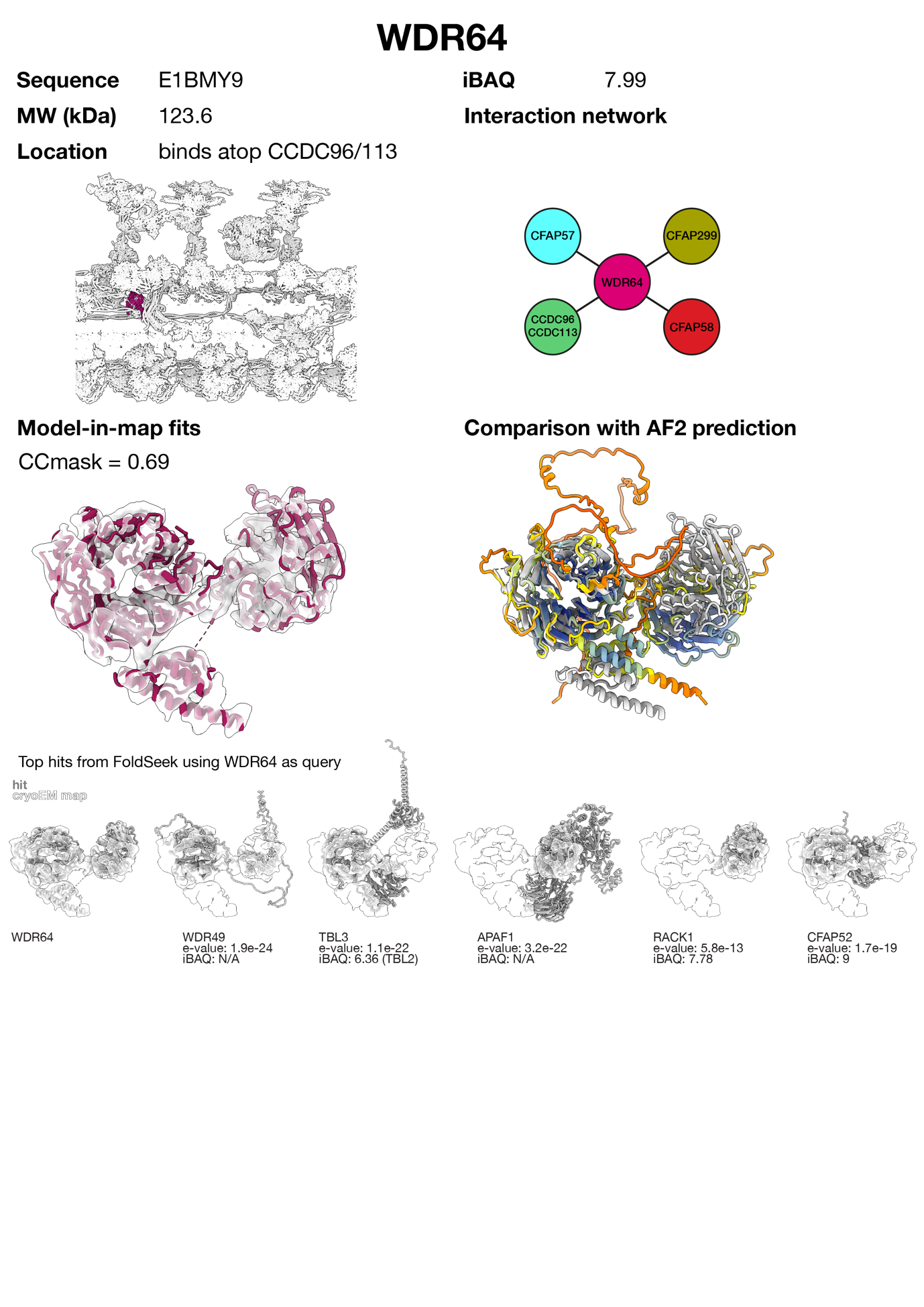


**Supplementary Figure 33 | Protein identification and assessment report for WDR64.** The AlphaFold prediction for WDR64 matches the cryo-EM density, i.e. two β-propellers and an EF-hand-like domain. An orthologous protein, CFAP337, binds to the same location in *Tetrahymena* axonemes^37^. Structurally similar proteins, including WDR49, were discarded because they did not match the density as well as WDR64 and/or were less abundant/not detected in the bovine sperm proteome.

**Supplementary Table 1 | Cryo-EM and cryo-ET data collection and processing statistics.** Reconstructions for the 96-nm DMT repeat from bovine sperm, bovine oviductal cilia, human oviductal cilia, and porcine brain ventricle cilia were obtained by single-particle analysis (SPA), while the reconstruction for porcine oviductal cilia was obtained by subtomogram averaging (STA). For SPA reconstructions, accession codes for composite maps are provided here; those for local refinements are provided in Supplementary Table 2.

|  | **Bovine sperm**  **(EMD-50664 and PDB 9FQR)** | **Bovine oviduct**  **(EMD-45783)** | **Human oviduct**  **(EMD-45785)** | **Porcine oviduct**  **(EMD-45677 to -45680)** | **Porcine brain ventricle**  **(EMD-45784)** |
| --- | --- | --- | --- | --- | --- |
| **Data collection and processing** | | | | | |
| **Method** | SPA | SPA | SPA | STA | SPA |
| **Facility*** | NeCEN & UU-EMC | CWRU | HC2EM | HC2EM | CWRU |
| **Microscope(s)** | Titan Krios (NeCEN) Talos Arctica (UU-EMC) | Titan Krios | Titan Krios | Titan Krios | Titan Krios |
| **Accelerating voltage (kV)** | 300 (Krios) 200 (Arctica) | 300 | 300 | 300 | 300 |
| **Energy filter** | BioQuantum | BioQuantum | BioQuantum | BioQuantum | BioQuantum |
| **Detector** | K3 (Krios) K2 (Arctica) | K3 | K3 | K3 | K3 |
| **Nominal magnification** | 81 000 (Krios)  130 000 (Arctica) | 64,000 | 81,000 | 53,000 | 64,000 |
| **Electron exposure (*e^-^*/Å^2^)** | 50 | 34 | 50 | 114 (over a range of ±54° in 3° steps, with 10 frames/tilt) | 34 |
| **Defocus range (µm)** | -0.5 to -2.5 | -0.5 to -2.0 | -0.8 to -2.0 | -2.0 to -4.0 | -0.5 to -2.0 |
| **Pixel size (Å)** | 1.06 (Krios)  1.041 (Arctica) | 1.34 | 1.06 | 1.68 | 1.34 |
| **Symmetry** | C1 | C1 | C1 | C1 | C1 |
| **Initial movie stacks (#)** | 19,070 (Krios) 26,361 (Arctica) | 4,716 | 21,348 | 137 (tilt series) | 7,051 |
| **Initial 8-nm particles (#)** | 2,585,140 | 1,115,389 | 1,627,650 | 128,356 | 1,256,289 |
| **Final 96-nm particles (#)** | 203,560 | 81,594 | 125,346 | 7,126 | 97,261 |
| **Resolution range (Å)** | See Supplementary Table 2 | | | 9.2-10.2 (at bin2) | See Sup Table 2 |
| **FSC threshold** | 0.143 | 0.143 | 0.143 | 0.143 | 0.143 |

*Abbreviations: NeCEN, Netherlands Center for Electron Nanoscopy. UU-EMC, Utrecht University EM Centre (UU-EMC). CWRU, Case Western Reserve University. HC2EM, Harvard Cryo-Electron Microscopy Center for Structural Biology.

**Supplementary Table 2 | Resolution ranges and EMDB IDs for locally refined regions of the 96-nm repeat analyzed by single particle analysis.** Resolution was estimated in cryoSPARC using the FSC = 0.143 criterion.

|  | **Resolution range (Å) and EMDB IDs** | | | |
| --- | --- | --- | --- | --- |
| **Region** | **Bovine sperm** | **Bovine oviduct** | **Human oviduct** | **Porcine brain ventricle** |
| DMT (tubulin) | 3.1-3.5 | 3.1-3.6 | 3-3.5 | 3.2-3.8 |
| RS1 | 3.9-5.1  EMD-50866 (RS1 base)  EMD-50867 (RS1 stalk)  EMD-50868 (RS1 head) | 6.6-9.0  EMD-45692 (RS1 base + stalk)  EMD-45693 (RS1 stalk + head) | 6.7-11.0  EMD- 45720 (RS1 base + stalk)  EMD- 45721 (RS1 stalk + head) | 7.3-9.0  EMD-45708 (RS1 base + stalk)  EMD-45709 (RS1 stalk + head) |
| TRiC | 7.4  EMD-50875 | - | - | - |
| RS2 | 3.8-4.5  EMD-50869 (RS2 base)  EMD-50870 (RS2 stalk)  EMD-50871 (RS2 head) | 8.0-9.2  EMD-45694 (RS2 base + stalk)  EMD-45695 (RS2 stalk + head) | 7.5-13.0  EMD- 45722 (RS2 base + stalk)  EMD- 45723 (RS2 stalk + head) | 7.1-8.8  EMD-45710 (RS2 base + stalk)  EMD-45711 (RS2 stalk + head) |
| RS3 | 4.1-4.9  EMD-50872 (RS3 base)  EMD-50873 (RS3 stalk)  EMD-50874 (RS3 head) | 5.9-10.0  EMD-45696 (RS3 base + stalk)  EMD-45697 (RS3 stalk + head) | 6.6-11.0  EMD- 45724 (RS3 base + stalk)  EMD- 45725 (RS3 stalk + head) | 6.7-9.7  EMD- 45712 (RS3 base + stalk)  EMD- 45713 (RS3 stalk + head) |
| RS2-RS3 bridge | 7.0  EMD-50876 | - | - | - |
| N-DRC | 3.5-4.7  EMD-50877 (baseplate)  EMD-50878 (distal lobe) | 3.9-9.3  EMD-45687 (baseplate)  EMD-45686 (distal lobe) | 3.8-6.8  EMD-45715 (baseplate)  EMD-45714 (distal lobe) | 3.8-9.3  EMD-45703 (baseplate)  EMD-45702 (distal lobe) |
| IDA*ab* | 3.9-6.7  EMD-50879 | 6.7 |  | 6.8 |
| IDA*c* | 4.0-5.3  EMD-50880 | 7.9  EMD-45683 | 6.8 | 7.8  EMD-45699 |
| IDA*e* | 4.3-5.3  EMD-50881 | 5.5-8.8  EMD-45684 | 7.0 | 6.6-8.6  EMD-45700 |
| IDA*dg* | 4.4-6.2 | 6.4-7.3 | 7.8 | 8.0 |
| IDA*f* IC/LC | 8-9  EMD-50882 (IC/LC) | 8.8  EMD-45685 | 9.3  EMD-45790 | 9.3 |
| IDA*f* motors | 4.6-5.9  EMD-50883 (part 1)  EMD-50884 (part 2) | 7.6-7.8  EMD-45690 (part 1)  EMD-45691 (part 2) | 6.9-7.8  EMD-45718 (part 1)  EMD-45719 (part 2) | 8.1-8.7  EMD-45706 (part 1)  EMD-45707 (part 2) |
| ODA-DC | 4.2  EMD-50885 | 3.6  EMD-45689 | 3.5  EMD-45717 | 3.9  EMD-45705 |
| ODA | 7.1  EMD-50886 | 8.7  EMD-45688 | 8.0  EMD-45716 | 8.7  EMD-45688 |

**Supplementary Table 3 | Summary of mass spectrometry instrumentation used to acquire the proteomics datasets analyzed in this study.** References are provided for previously reported datasets that were reanalyzed here. The total number of proteins identified in each dataset is also reported.

| **Cilia Type** | **Sperm** | | | | **Oviduct** | | **Brain ventricle** | **Respiratory tract** | |
| --- | --- | --- | --- | --- | --- | --- | --- | --- | --- |
| **Species** | **Bull** | **Pig** | **Mouse** | **Human** | **Bull** | **Human** | **Pig** | **Bull** | **Human** |
| **Reference** | Leung et al 2023 ^38^ | Leung et al 2021 ^39^ | Zhou et al 2023 ^40^ | Zhou et al 2023 ^40^ | This study | This study | This study | Gui et al 2021 ^41^ | Gui et al 2022 ^42^ |
| **Instrument** | Orbitrap Lumos | Q Exactive HF-X | Q Exactive HF | Q Exactive HF | Orbitrap Eclipse | Orbitrap Velos Pro | Orbitrap Eclipse | Orbitrap Velos Pro | Orbitrap Velos Pro |
| **Number of proteins identified** | 4402 | 2069 | 4303 | 1954 | 849 | 1713 | 4000 | 650 | 745 |

**Supplementary Table 4 | Proteomics data for various mammalian motile cilia.** Proteomics data for bovine oviduct cilia, human oviduct cilia and porcine brain ventricle cilia were newly collected for this study. Previously published proteomics data for bovine respiratory cilia^41^, human respiratory cilia^42^, bovine sperm^38^, mouse sperm^40^, porcine sperm^39^ and human sperm^40^ were re-analyzed in this study and are also presented.

**Supplementary Table 5 | Refinement and validation statistics for atomic models reported in this study.** Due to the size of the model, the 96-nm repeat was split into two halves that were refined independently. Statistics represent the average of these refinements. Motor domains of ODAs and IDAs other than IDA*a* and IDA*f* were rigid-body fit into the maps and not refined as these regions are of lower resolution; hence, they are not included in the refinement statistics.

|  | **96-nm DMT repeat from bovine sperm (PDB 9FQR)** | **48-nm DMT repeat from bovine oviductal cilia**  **(PDB 9CPB)** | **48-nm DMT repeat from porcine brain ventricle cilia**  **(PDB 9CPC)** |
| --- | --- | --- | --- |
| **Resolution limit set in refinement (Å)** | 5 | 3.5 | 3.7 |
| **Correlation coefficient (CCmask)** | 0.65 | 0.70 | 0.65 |
| **Model composition** |  |  |  |
| Non-H atoms | 4,326,255 | 1,132,899 | 1,114,870 |
| Protein residues | 539,967 | 142,674 | 140,471 |
| Ligands | GDP: 362 GTP: 359 | GDP: 130  GTP: 134 | GDP: 133  GTP: 136 |
| **B-factors (Å^2^)** |  |  |  |
| Protein | 98.63 | 89.83 | 77.42 |
| Ligand | 47.7 | 90.82 | 92.13 |
| **RMS deviations** |  |  |  |
| Bond length (Å) | 0.008 | 0.010 | 0.007 |
| Bond angles (°) | 1.19 | 1.327 | 1.142 |
| **Validation** |  |  |  |
| MolProbity score | 2.12 | 2.11 | 2.09 |
| Clashscore | 9.7 | 11.94 | 11.72 |
| Poor rotamers (%) | 0.07 | 0.06 | 0.67 |
| **Ramachandran plot** |  |  |  |
| Favored (%) | 94.57 | 90.88 | 91.50 |
| Allowed (%) | 5.3 | 8.71 | 7.92 |
| Disallowed (%) | 0.14 | 0.40 | 0.58 |

**Supplementary Table 6 | Newly identified axonemal proteins and their distribution across mammalian motile cilium types.** Distribution is inferred from proteomics data reported in this study and in previous work, along with expression data from the Human Protein Atlas. This table also reports sequence identifiers of putative orthologs in *Chlamydomonas reinhardtii*, if any. Colors in this table (blue for proteins present in all motile cilia and pink for sperm-specific proteins) correspond to the colors in Figure 2a.

**Supplementary Table 7 | Summary of disease phenotypes associated with disruption of axonemal proteins.** Colors in this table correspond to the colors in Figure 2. References cited in this table: TUBB4B ^43^, CCDC39 ^44–51^, CCDC40 ^49,50,52–55^, CFAP58 ^56,57^, CCDC146 ^58,59^, AK8 ^7,60^, CAMK4 ^61–63^, CFAP206 ^26,64^, CYB5D1 ^65^, DNAJB13 ^66–69^, IQUB ^8,70^, LRRC34 ^71^, NME5 ^60,72,73^, ROPN1L ^74^, RSPH1 ^49,50,75–78^, RSPH3 ^50,79,80^, RSPH6A ^81–83^, RSPH9 ^50,84–87^, AKAP14 ^88^, AK7 ^2,60,89–92^, AK9 ^11,12,93^, CATIP ^21,94^, CFAP91 ^95–98^, CFAP251 ^99–101^, LRRC23 ^13,29,102,103^, PRKACA ^104–106^, PRKAR1A/1B ^107,108^, STKLD1 ^109^, STYXL1 ^23^, ANKEF1 ^110^, DRC1 ^111–115^, DRC2 ^116–120^, DRC3 ^121,122^, DRC4 ^123–126^, DRC5 ^127,128^, DRC7 ^129^, DRC9 ^130,131^, DRC10 ^132^, DNAH8 ^133–135^, DNAH17 ^136–140^, DNAI1 ^141,142^, DNAI2 ^143–145^, CCDC63 ^146^, ODAD1 ^147,148^, ODAD2 ^149–151^, ODAD3 ^152–155^, ODAD4 ^156,157^, ODAD5 ^158,159^, CFAP57 ^160,161^, DNAH2 ^162–165^, DNAH10 ^166–169^, DNAI3 ^170,171^, DNAI4 ^165^, DYNLL1 ^172^, DYNLT2B ^173^, CCDC39 ^174–178^, CFAP44 ^174–177^, DNAH1 ^177,179–187^, DNAH3 ^188^, DNAH6 ^189–191^, DNAH7 ^192–194^, DNAH12 ^195–197^, DNALI1 ^198–203^, CETN1 ^204,205^, TTC29 ^206,207^, ZMYND12 ^208^.

**Supplementary Table 8 | Expression clusters of core and sperm-specific axonemal proteins during spermatogenesis.** Data were retrieved from ^209^, a single-cell RNA-sequencing study that analyzed gene expression in three distinct mouse cell types (spermatogonia, spermatocytes, and round spermatids), each representing a unique stage of spermatogenesis. The genes were subsequently clustered into six classes, numbered 1-6, based on their relative RNA-sequencing signal within each cell type. Classes with lower numbers (e.g. 1 and 2) include genes predominantly expressed in spermatogonia (and therefore early in spermatogenesis) whereas classes with higher numbers (e.g. 5 and 6) include genes most highly expressed in round spermatids, signifying their activation later in spermatogenesis.

**Supplementary Video 1 | Molecular composition and organization of radial spoke 3 (RS3) in bovine sperm.** Proteins are shown one at a time for clarity. Note that the order of appearance is not meant to imply order of assembly.

**Supplementary Video 2 | Conformational changes of inner dynein arm *f* (IDA*f*) associated with the transition from post-stroke to pre-stroke state.** Video shows morphs between models of the post-stroke state PDB 8J07^210^ and the pre-stroke state (this study).

**Supplementary Video 3 | Conformational changes of outer dynein arms (ODAs) associated with the transition from post-stroke to pre-stroke state.** Video shows morphs between models of the post-stroke state (PDB 8J07^210^) and the pre-stroke state (this study).

**Supplementary References**

1. Chen, Z. *et al.* De novo protein identification in mammalian sperm using in situ cryoelectron tomography and AlphaFold2 docking. *Cell* **186**, 5041-5053.e19 (2023).

2. Lorès, P. *et al.* Homozygous missense mutation L673P in adenylate kinase 7 (AK7) leads to primary male infertility and multiple morphological anomalies of the flagella but not to primary ciliary dyskinesia. *Hum Mol Genet* **27**, 1196–1211 (2018).

3. Sheridan, J. *et al.* Novel centriolar defects underlie a primary ciliary dyskinesia phenotype in an adenylate kinase 7 deficient ciliated epithelium . Northwestern University , Feinberg School of Medicine , Department of Cell and Northwestern University , Lurie Cancer Center. *bioRxiv* (2023) doi:10.1101/2023.07.25.550535.

4. Urbanska, P. *et al.* The CSC proteins FAP61 and FAP251 build the basal substructures of radial spoke 3 in cilia. *Mol Biol Cell* **26**, 1463–1475 (2015).

5. Bicka, M. *et al.* Heterogeneity of radial spokes structural components and associated enzymes in Tetrahymena cilia. *Elife* 1–49 (2023) doi:10.7554/eLife.90003.1.

6. Vadnais, M. L. *et al.* Adenine nucleotide metabolism and a role for AMP in modulating flagellar waveforms in mouse sperm. *Biol Reprod* **90**, 1–14 (2014).

7. Wu, H. *et al.* Adenylate kinase phosphate energy shuttle underlies energetic communication in flagellar axonemes. *Sci China Life Sci* (2024) doi:10.1007/s11427-023-2539-1.

8. Zhang, X. *et al.* Differential requirements of IQUB for the assembly of radial spoke 1 and the motility of mouse cilia and flagella. *Cell Rep* **41**, 111683 (2022).

9. Meng, X. *et al.* Multi-scale structures of the mammalian radial spoke and divergence of axonemal complexes in ependymal cilia. *Nat Commun* **15**, (2024).

10. McCafferty, C. L. *et al.* An amino acid-resolution interactome for motile cilia illuminates the structure and function of ciliopathy protein complexes. *bioRxiv* 2023.07.09.548259 (2023).

11. Yang, H. W. *et al.* A role for mutations in AK9 and other genes affecting ependymal cells in idiopathic normal pressure hydrocephalus. *Proceedings of the National Academy of Sciences* **120**, 2017 (2023).

12. Sha, Y. *et al.* Deficiency in AK9 causes asthenozoospermia and male infertility by destabilising sperm nucleotide homeostasis. *EBioMedicine* **96**, 104798 (2023).

13. Hwang, J. Y. *et al.* LRRC23 truncation impairs radial spoke 3 head assembly and sperm motility underlying male infertility. *Elife* **12**, 1–39 (2023).

14. Kultgen, P. L., Byrd, S. K., Ostrowski, L. E. & Milgram, S. L. Characterization of an A-Kinase Anchoring Protein in Human Ciliary Axonemes. *Mol Biol Cell* **13**, 4156–4166 (2002).

15. Uhlén, M. *et al.* Tissue-based map of the human proteome. *Science (1979)* **347**, (2015).

16. Daniel, J. G. & Panizzi, J. R. Spatiotemporal expression profile of embryonic and adult ankyrin repeat and EF-hand domain containing protein 1-encoding genes ankef1a and ankef1b in zebrafish. *Gene Expression Patterns* **34**, (2019).

17. Erickson, T., Biggers, W. P., Williams, K., Butland, S. E. & Venuto, A. Regionalized Protein Localization Domains in the Zebrafish Hair Cell Kinocilium. *J Dev Biol* **11**, (2023).

18. Marín-Briggiler, C. I. *et al.* Evidence of the presence of calcium/calmodulin-dependent protein kinase IV in human sperm and its involvement in motility regulation. *J Cell Sci* **118**, 2013–2022 (2005).

19. Nguyen, T. M. D., Combarnous, Y., Praud, C., Duittoz, A. & Blesbois, E. Ca2+/calmodulin-dependent protein kinase kinases (CaMKKs) effects on AMP-activated protein kinase (AMPK) regulation of chicken sperm functions. *PLoS One* **11**, 1–22 (2016).

20. Morita, M., Takemura, A., Nakajima, A. & Okuno, M. Microtubule sliding movement in tilapia sperm flagella axoneme is regulated by Ca2+/calmodulin-dependent protein phosphorylation. *Cell Motil Cytoskeleton* **63**, 459–470 (2006).

21. Bontems, F. *et al.* C2orf62 and TTC17 are involved in actin organization and ciliogenesis in zebrafish and human. *PLoS One* **9**, (2014).

22. Redgrove, K. A. *et al.* Involvement of multimeric protein complexes in mediating the capacitation-dependent binding of human spermatozoa to homologous zonae pellucidae. *Dev Biol* **356**, 460–474 (2011).

23. Chen, Y. *et al.* STYXL1 regulates CCT complex assembly and flagellar tubulin folding in sperm formation. *Nat Commun* **15**, 1–15 (2024).

24. Liu, C. *et al.* Pathway and mechanism of tubulin folding mediated by TRiC/CCT along its ATPase cycle revealed using cryo-EM. *Commun Biol* **6**, 1–14 (2023).

25. Han, W. *et al.* Structural basis of plp2-mediated cytoskeletal protein folding by TRiC/CCT. *Sci Adv* **9**, 1–14 (2023).

26. Beckers, A. *et al.* *The FOXJ1 Target Cfap206 Is Required for Sperm Motility, Mucociliary Clearance of the Airways and Brain Development*. *Development (Cambridge)* vol. 147 (2020).

27. Vasudevan, K. K. *et al.* FAP206 is a microtubule-docking adapter for ciliary radial spoke 2 and dynein c. *Mol Biol Cell* **26**, 696–710 (2015).

28. Jamali, K. *et al.* Automated model building and protein identification in cryo-EM maps. *Nature* (2024) doi:10.1038/s41586-024-07215-4.

29. Zhang, X. *et al.* *LRRC23 Is a Conserved Component of the Radial Spoke That Is Necessary for Sperm Motility and Male Fertility in Mice*. *Journal of cell science* vol. 134 (2021).

30. Stival, C. *et al.* Disruption of protein kinase A localization induces acrosomal exocytosis in capacitated mouse sperm. *Journal of Biological Chemistry* **293**, 9435–9447 (2018).

31. Xu, K., Yang, L., Zhang, L. & Qi, H. Lack of AKAP3 disrupts integrity of the subcellular structure and proteome of mouse sperm and causes male sterility. *Development* **147**, dev181057 (2020).

32. Wertheimer, E. *et al.* Compartmentalization of distinct cAMP signaling pathways in mammalian sperm. *Journal of Biological Chemistry* **288**, 35307–35320 (2013).

33. Zapata-Carmona, H. *et al.* The activation of the chymotrypsin-like activity of the proteasome is regulated by soluble adenyl cyclase/cAMP/protein kinase A pathway and required for human sperm capacitation. *Mol Hum Reprod* **25**, 587–600 (2019).

34. Porter, M. E. & Sale, W. S. The 9 + 2 axoneme anchors multiple inner arm dyneins and a network of kinases and phosphatases that control motility. *Journal of Cell Biology* **151**, 37–42 (2000).

35. Walton, T. *et al.* Axonemal structures reveal mechanoregulatory and disease mechanisms. *Nature* **618**, 625–633 (2023).

36. Beneke, T., Banecki, K., Fochler, S. & Gluenz, E. LAX28 is required for stable assembly of the inner dynein arm f/l1 and tether/tether head complex in Leishmania flagella. *J Cell Sci* **133**, (2020).

37. Ghanaeian, A. *et al.* Integrated modeling of the Nexin-dynein regulatory complex reveals its regulatory mechanism. *Nat Commun* **14**, (2023).

38. Leung, M. R. *et al.* Structural specializations of the sperm tail. *Cell* **186**, 2880-2896.e17 (2023).

39. Leung, M. R. *et al.* In-cell structures of conserved supramolecular protein arrays at the mitochondria–cytoskeleton interface in mammalian sperm. *Proceedings of the National Academy of Sciences* **118**, 1–10 (2021).

40. Zhou, L. *et al.* Structures of sperm flagellar doublet microtubules expand the genetic spectrum of male infertility. *Cell* **186**, 2897-2910.e19 (2023).

41. Gui, M. *et al.* De novo identification of mammalian ciliary motility proteins using cryo-EM. *Cell* **184**, 5791-5806.e19 (2021).

42. Gui, M. *et al.* SPACA9 is a lumenal protein of human ciliary singlet and doublet microtubules. *Proc Natl Acad Sci U S A* **119**, e2207605119 (2022).

43. Dodd, D. O. *et al.* Ciliopathy patient variants reveal organelle-specific functions for TUBB4B in axonemal microtubules. *Science (1979)* **384**, (2024).

44. Merveille, A. C. *et al.* CCDC39 is required for assembly of inner dynein arms and the dynein regulatory complex and for normal ciliary motility in humans and dogs. *Nat Genet* **43**, 72–78 (2011).

45. Abo, M. *et al.* Primary ciliary dyskinesia with CCDC39 variants displaying specific ciliary ultrastructure and movement concordant with the genotype: A case report. *Respir Investig* **60**, 725–728 (2022).

46. Chen, D. *et al.* A novel CCDC39 mutation causes multiple morphological abnormalities of the flagella in a primary ciliary dyskinesia patient. *Reprod Biomed Online* **43**, 920–930 (2021).

47. Antony, D. *et al.* Mutations in CCDC39 and CCDC40 are the Major Cause of Primary Ciliary Dyskinesia with Axonemal Disorganization and Absent Inner Dynein Arms. *Hum Mutat* **34**, 462–472 (2013).

48. Abdelhamed, Z. *et al.* A mutation in Ccdc39 causes neonatal hydrocephalus with abnormal motile cilia development in mice. *Development (Cambridge)* **145**, (2018).

49. Aprea, I. *et al.* Pathogenic gene variants in CCDC39, CCDC40, RSPH1, RSPH9, HYDIN, and SPEF2 cause defects of sperm flagella composition and male infertility. *Front Genet* **14**, (2023).

50. Vanaken, G. J. *et al.* Infertility in an adult cohort with primary ciliary dyskinesia: Phenotype-gene association. *European Respiratory Journal* **50**, 61–64 (2017).

51. Cannarella, R. *et al.* Ultrastructural Sperm Flagellum Defects in a Patient With CCDC39 Compound Heterozygous Mutations and Primary Ciliary Dyskinesia/Situs Viscerum Inversus. *Front Genet* **11**, 1–14 (2020).

52. Becker-Heck, A. *et al.* The coiled-coil domain containing protein CCDC40 is essential for motile cilia function and left-right axis formation. *Nat Genet* **43**, 79–84 (2011).

53. Liu, L., Zhou, K., Song, Y. & Liu, X. CCDC40 mutation as a cause of infertility in a Chinese family with primary ciliary dyskinesia. *Medicine (United States)* **100**, E28275 (2021).

54. Yang, L. *et al.* Compound heterozygous variants in the Coiled-Coil Domain Containing 40 gene in a chinese family with primary ciliary dyskinesia cause extreme phenotypic diversity in cilia ultrastructure. *Front Genet* **9**, 1–10 (2018).

55. Sui, W. *et al.* CCDC40 mutation as a cause of primary ciliary dyskinesia: a case report and review of literature. *Clin Respir J* **10**, 614–621 (2016).

56. He, X. *et al.* Bi-allelic Loss-of-function Variants in CFAP58 Cause Flagellar Axoneme and Mitochondrial Sheath Defects and Asthenoteratozoospermia in Humans and Mice. *Am J Hum Genet* **107**, 514–526 (2020).

57. Sha, Y. *et al.* Biallelic mutations of CFAP58 are associated with multiple morphological abnormalities of the sperm flagella. *Clin Genet* **99**, 443–448 (2021).

58. Muroňová, J. *et al.* Lack of CCDC146, a ubiquitous centriole and microtubule-associated protein, leads to non-syndromic male infertility in human and mouse. *Elife* **13**, 1–44 (2024).

59. Ma, Y. *et al.* CCDC146 is required for sperm flagellum biogenesis and male fertility in mice. *Cellular and Molecular Life Sciences* **81**, (2024).

60. Vogel, P. *et al.* Congenital hydrocephalus in genetically engineered mice. *Vet Pathol* **49**, 166–181 (2012).

61. Wu, J. Y. *et al.* Spermiogenesis and exchange of basic nuclear proteins are impaired in male germ cells lacking Camk4. *Nat Genet* **25**, 448–452 (2000).

62. Wu, J. Y., Gonzalez-Robayna, I. J., Richards, J. S. & Means, A. R. Female fertility is reduced in mice lacking Ca2+/calmodulin-dependent protein kinase IV. *Endocrinology* **141**, 4777–4783 (2000).

63. Khattri, A. *et al.* Novel mutations in calcium/calmodulin-dependent protein kinase IV (CAMK4) gene in infertile men. *Int J Androl* **35**, 810–818 (2012).

64. Shen, Q. *et al.* Bi-allelic truncating variants in CFAP206 cause male infertility in human and mouse. *Hum Genet* **140**, 1367–1377 (2021).

65. Zhao, L. *et al.* Heme-binding protein CYB5D1 is a radial spoke component required for coordinated ciliary beating. *Proc Natl Acad Sci U S A* **118**, 1–12 (2021).

66. Liu, M. *et al.* A novel homozygous mutation in DNAJB13—a gene associated with the sperm axoneme—leads to teratozoospermia. *J Assist Reprod Genet* **39**, 757–764 (2022).

67. Oji, A. *et al.* CRISPR/Cas9 mediated genome editing in ES cells and its application for chimeric analysis in mice. *Sci Rep* **6**, 1–9 (2016).

68. El Khouri, E. *et al.* Mutations in DNAJB13, Encoding an HSP40 Family Member, Cause Primary Ciliary Dyskinesia and Male Infertility. *Am J Hum Genet* **99**, 489–500 (2016).

69. Li, W. N. *et al.* Missense mutation in DNAJB13 gene correlated with male fertility in asthenozoospermia. *Andrology* **8**, 299–306 (2020).

70. Zhang, Z. *et al.* IQUB deficiency causes male infertility by affecting the activity of p-ERK1/2/RSPH3. *Human Reproduction* **38**, 168–179 (2023).

71. Shamseldin, H. E. *et al.* The morbid genome of ciliopathies: an update. *Genetics in Medicine* **22**, 1051–1060 (2020).

72. Cho, E. H. *et al.* A nonsense variant in NME5 causes human primary ciliary dyskinesia with radial spoke defects. *Clin Genet* **98**, 64–68 (2020).

73. Anderegg, L. *et al.* NME5 frameshift variant in Alaskan Malamutes with primary ciliary dyskinesia. *PLoS Genet* 1–16 (2019).

74. Fiedler, S. E., Dudiki, T., Vijayaraghavan, S. & Carr, D. W. Loss of R2D2 proteins ROPN1 and ROPN1L causes defects in murine sperm motility, phosphorylation, and fibrous sheath integrity. *Biol Reprod* **88**, 1–10 (2013).

75. Knowles, M. R. *et al.* Mutations in RSPH1 cause primary ciliary dyskinesia with a unique clinical and ciliary phenotype. *Am J Respir Crit Care Med* **189**, 707–717 (2014).

76. Onoufriadis, A. *et al.* Targeted NGS gene panel identifies mutations in RSPH1 causing primary ciliary dyskinesia and a common mechanism for ciliary central pair agenesis due to radial spoke defects. *Hum Mol Genet* **23**, 3362–3374 (2014).

77. Yin, W. *et al.* Mice with a deletion of Rsph1 exhibit a low level of mucociliary clearance and develop a primary ciliary dyskinesia phenotype. *Am J Respir Cell Mol Biol* **61**, 312–321 (2019).

78. Kott, E. *et al.* Loss-of-function mutations in RSPH1 Cause primary ciliary dyskinesia with central-complex and radial-spoke defects. *Am J Hum Genet* **93**, 561–570 (2013).

79. Jeanson, L. *et al.* RSPH3 Mutations Cause Primary Ciliary Dyskinesia with Central-Complex Defects and a Near Absence of Radial Spokes. *Am J Hum Genet* **97**, 153–162 (2015).

80. Wu, H. *et al.* Patients with severe asthenoteratospermia carrying SPAG6 or RSPH3 mutations have a positive pregnancy outcome following intracytoplasmic sperm injection. *J Assist Reprod Genet* **37**, 829–840 (2020).

81. Abbasi, F. *et al.* RSPH6A is required for sperm flagellum formation and male fertility in mice. *J Cell Sci* **131**, (2018).

82. De Jesús-Rojas, W. *et al.* The RSPH4A Gene in Primary Ciliary Dyskinesia. *Int J Mol Sci* **24**, (2023).

83. Yoke, H. *et al.* Rsph4a is essential for the triplet radial spoke head assembly of the mouse motile cilia. *PLoS Genet* **16**, 1–16 (2020).

84. Castleman, V. H. *et al.* Mutations in radial spoke head protein genes RSPH9 and RSPH4A cause primary ciliary dyskinesia with central-microtubular-pair abnormalities. *Am J Hum Genet* **84**, 197–209 (2008).

85. Yiallouros, P. K. *et al.* Wide phenotypic variability in RSPH9-associated primary ciliary dyskinesia: Review of a case-series from Cyprus. *J Thorac Dis* **11**, 2067–2075 (2019).

86. Al-Mutairi, D. A., Alsabah, B. H., Pennekamp, P. & Omran, H. Mapping the Most Common Founder Variant in RSPH9 That Causes Primary Ciliary Dyskinesia in Multiple Consanguineous Families of Bedouin Arabs. *J Clin Med* **12**, (2023).

87. Zou, W. *et al.* Loss of Rsph9 causes neonatal hydrocephalus with abnormal development of motile cilia in mice. *Sci Rep* **10**, 1–11 (2020).

88. Montjean, D. *et al.* Sperm transcriptome profiling in oligozoospermia. *J Assist Reprod Genet* **29**, 3–10 (2012).

89. Chang, T. *et al.* A novel homozygous nonsense variant of AK7 is associated with multiple morphological abnormalities of the sperm flagella. *Reprod Biomed Online* **48**, 1–11 (2024).

90. Fernandez-Gonzalez, A., Kourembanas, S., Wyatt, T. A. & Mitsialis, S. A. Mutation of murine adenylate kinase 7 underlies a primary ciliary dyskinesia phenotype. *Am J Respir Cell Mol Biol* **40**, 305–313 (2009).

91. Mata, M. *et al.* New adenylate kinase 7 (AK7) mutation in primary ciliary dyskinesia. *Am J Rhinol Allergy* **26**, 260–264 (2012).

92. Milara, J., Armengot, M., Mata, M., Morcillo, E. J. & Cortijo, J. Role of adenylate kinase type 7 expression on cilia motility: Possible link in primary ciliary dyskinesia. *Am J Rhinol Allergy* **24**, 181–185 (2010).

93. O’Callaghan, E. *et al.* Adenylate kinase 9 is essential for sperm function and male fertility in mammals. *Proceedings of the National Academy of Sciences* **120**, 2017 (2023).

94. Arafat, M. *et al.* Mutation in CATIP (C2orf62) causes oligoteratoasthenozoospermia by affecting actin dynamics. *J Med Genet* **58**, 106–115 (2021).

95. Hu, T. *et al.* Biallelic CFAP61 variants cause male infertility in humans and mice with severe oligoasthenoteratozoospermia. *J Med Genet* **60**, 144–153 (2023).

96. Huang, T. *et al.* Absence of murine CFAP61 causes male infertility due to multiple morphological abnormalities of the flagella. *Sci Bull (Beijing)* **65**, 854–864 (2020).

97. Liu, S. *et al.* CFAP61 is required for sperm flagellum formation and male fertility in human and mouse. *Development (Cambridge)* **148**, (2021).

98. Ma, A. *et al.* Biallelic Variants in CFAP61 Cause Multiple Morphological Abnormalities of the Flagella and Male Infertility. *Front Cell Dev Biol* **9**, 1–9 (2022).

99. Kherraf, Z. E. *et al.* A Homozygous Ancestral SVA-Insertion-Mediated Deletion in WDR66 Induces Multiple Morphological Abnormalities of the Sperm Flagellum and Male Infertility. *Am J Hum Genet* **103**, 400–412 (2018).

100. Li, W. *et al.* Biallelic mutations of CFAP251 cause sperm flagellar defects and human male infertility. *J Hum Genet* **64**, 49–54 (2019).

101. Auguste, Y. *et al.* Loss of Calmodulin- and Radial-Spoke-Associated Complex Protein CFAP251 Leads to Immotile Spermatozoa Lacking Mitochondria and Infertility in Men. *Am J Hum Genet* **103**, 413–420 (2018).

102. Li, Y. *et al.* LRRC23 deficiency causes male infertility with idiopathic asthenozoospermia by disrupting the assembly of radial spokes. *Clin Genet* **104**, 694–699 (2023).

103. Han, X., Xie, H., Wang, Y. & Zhao, C. Radial spoke proteins regulate otolith formation during early zebrafish development. *FASEB Journal* **32**, 3984–3992 (2018).

104. Skålhegg, B. S. *et al.* Mutation of the Cα subunit of PKA leads to growth retardation and sperm dysfunction. *Molecular Endocrinology* **16**, 630–639 (2002).

105. Palencia-campos, A. *et al.* Germline and Mosaic Variants in PRKACA and PRKACB Cause a Multiple Congenital Malformation Syndrome. 977–988 (2020) doi:10.1016/j.ajhg.2020.09.005.

106. Nolan, M. A. *et al.* Sperm-specific protein kinase A catalytic subunit Cα2 orchestrates cAMP signaling for male fertility. *Proc Natl Acad Sci U S A* **101**, 13483–13488 (2004).

107. Burton, K. A. *et al.* Haploinsufficiency at the protein kinase A RIα gene locus leads to fertility defects in male mice and men. *Molecular Endocrinology* **20**, 2504–2513 (2006).

108. Veugelers, M. *et al.* Comparative PRKAR1A genotype-phenotype analyses in humans with Carney complex and prkar1a haploinsufficient mice. *Proc Natl Acad Sci U S A* **101**, 14222–14227 (2004).

109. Umair, M. *et al.* Whole-exome sequencing revealed a nonsense mutation in STKLD1 causing non-syndromic pre-axial polydactyly type A affecting only upper limb. *Clin Genet* **96**, 134–139 (2019).

110. Canon, C. M., Buyuk, E., Copperman, A. B., García-Sastre, A. & Miorin, L. THE EVOLUTIONARILY CONSERVED PROTEIN ANKEF1 CONFERS SPERM MIDPIECE FLEXIBILITY AND IS ESSENTIAL FOR MALE FERTILITY. *Fertil Steril* **120**, e134 (2023).

111. Lei, C. *et al.* DRC1 deficiency caused primary ciliary dyskinesia and MMAF in a Chinese patient. *J Hum Genet* **67**, 197–201 (2022).

112. Morimoto, K. *et al.* Recurring large deletion in DRC1 (CCDC164) identified as causing primary ciliary dyskinesia in two Asian patients. *Mol Genet Genomic Med* **7**, 3–7 (2019).

113. Liu, Y. *et al.* Case Report: Whole-Exome Sequencing-Based Copy Number Variation Analysis Identified a Novel DRC1 Homozygous Exon Deletion in a Patient With Primary Ciliary Dyskinesia. *Front Genet* **13**, 1–9 (2022).

114. Wirschell, M. *et al.* The nexin-dynein regulatory complex subunit DRC1 is essential for motile cilia function in algae and humans. *Nat Genet* **45**, 262–268 (2013).

115. Pereira, R. *et al.* Characterization of a DRC1 null variant associated with primary ciliary dyskinesia and female infertility. *J Assist Reprod Genet* **40**, 765–778 (2023).

116. Zhang, Z. *et al.* CCDC65, a Gene Knockout that leads to Early Death of Mice, acts as a potentially Novel Tumor Suppressor in Lung Adenocarcinoma. *Int J Biol Sci* **18**, 4171–4186 (2022).

117. Austin-Tse, C. *et al.* Zebrafish ciliopathy screen plus human mutational analysis identifies C21orf59 and CCDC65 defects as causing primary ciliary dyskinesia. *Am J Hum Genet* **93**, 672–686 (2013).

118. Ben Braiek, M. *et al.* A Nonsense Variant in CCDC65 Gene Causes Respiratory Failure Associated with Increased Lamb Mortality in French Lacaune Dairy Sheep. *Genes (Basel)* **13**, 1–14 (2021).

119. Horani, A. *et al.* CCDC65 Mutation Causes Primary Ciliary Dyskinesia with Normal Ultrastructure and Hyperkinetic Cilia. *PLoS One* **8**, (2013).

120. Jreijiri, F. *et al.* CCDC65, encoding a component of the axonemal Nexin-Dynein regulatory complex, is required for sperm flagellum structure in humans. *Clin Genet* **105**, 317–322 (2024).

121. Ha, S., Lindsay, A. M., Timms, A. E. & Beier, D. R. Mutations in Dnaaf1 and Lrrc48 cause hydrocephalus, laterality defects, and sinusitis in mice. *G3: Genes, Genomes, Genetics* **6**, 2479–2487 (2016).

122. Zhou, S. *et al.* DRC3 is an assembly adapter of the nexin-dynein regulatory complex functional components during spermatogenesis in humans and mice. *Signal Transduct Target Ther* **8**, 1–4 (2023).

123. Colantonio, J. R. *et al.* The dynein regulatory complex is required for ciliary motility and otolith biogenesis in the inner ear. *Nature* **457**, 205–209 (2009).

124. Jeanson, L. *et al.* Mutations in GAS8, a Gene Encoding a Nexin-Dynein Regulatory Complex Subunit, Cause Primary Ciliary Dyskinesia with Axonemal Disorganization. *Hum Mutat* **37**, 776–785 (2016).

125. Olbrich, H. *et al.* Loss-of-Function GAS8 Mutations Cause Primary Ciliary Dyskinesia and Disrupt the Nexin-Dynein Regulatory Complex. *Am J Hum Genet* **97**, 546–554 (2015).

126. Kherraf, Z. E. *et al.* A splice donor variant of GAS8 induces structural disorganization of the axoneme in sperm flagella and leads to nonsyndromic male infertility. *Clin Genet* **105**, 220–225 (2024).

127. Zhou, S. *et al.* Bi-allelic variants in human TCTE1/DRC5 cause asthenospermia and male infertility. *European Journal of Human Genetics* **30**, 721–729 (2022).

128. Castaneda, J. M. *et al.* TCTE1 is a conserved component of the dynein regulatory complex and is required for motility and metabolism in mouse spermatozoa. *Proc Natl Acad Sci U S A* **114**, E5370–E5378 (2017).

129. Morohoshi, A. *et al.* Nexin-Dynein regulatory complex component DRC7 but not FBXL13 is required for sperm flagellum formation and male fertility in mice. *PLoS Genet* **16**, 1–21 (2020).

130. Li, R. K. *et al.* Iqcg is essential for sperm flagellum formation in mice. *PLoS One* **9**, 1–12 (2014).

131. Harris, T. P., Schimenti, K. J., Munroe, R. J. & Schimenti, J. C. IQ motif-containing G (Iqcg) is required for mouse spermiogenesis. *G3: Genes, Genomes, Genetics* **4**, 367–372 (2014).

132. Zhang, P., Jiang, W., Luo, N., Zhu, W. & Fan, L. IQ motif containing D (IQCD), a new acrosomal protein involved in the acrosome reaction and fertilisation. *Reprod Fertil Dev* **31**, 898 (2019).

133. Yang, Y. *et al.* Loss-of-function mutation in DNAH8 induces asthenoteratospermia associated with multiple morphological abnormalities of the sperm flagella. *Clin Genet* **98**, 396–401 (2020).

134. Weng, M. *et al.* Mutations in DNAH8 contribute to multiple morphological abnormalities of sperm flagella and male infertility. *Acta Biochim Biophys Sin (Shanghai)* **53**, 472–480 (2021).

135. Liu, C. *et al.* Bi-allelic DNAH8 Variants Lead to Multiple Morphological Abnormalities of the Sperm Flagella and Primary Male Infertility. *Am J Hum Genet* **107**, 330–341 (2020).

136. Nosková, A. *et al.* Infertility due to defective sperm flagella caused by an intronic deletion in DNAH17 that perturbs splicing. *Genetics* **217**, (2021).

137. Zhang, B. *et al.* A DNAH17 missense variant causes flagella destabilization and asthenozoospermia. *Journal of Experimental Medicine* **217**, (2020).

138. Sha, Y. *et al.* DNAH17 is associated with asthenozoospermia and multiple morphological abnormalities of sperm flagella. *Ann Hum Genet* **84**, 271–279 (2020).

139. Whitfield, M. *et al.* Mutations in DNAH17, Encoding a Sperm-Specific Axonemal Outer Dynein Arm Heavy Chain, Cause Isolated Male Infertility Due to Asthenozoospermia. *Am J Hum Genet* **105**, 198–212 (2019).

140. Liu, Z. *et al.* Novel compound heterozygous variants of DNAH17 in a Chinese infertile man with multiple morphological abnormalities of sperm flagella. *Andrologia* **54**, (2022).

141. Guichard, C. *et al.* Axonemal dynein intermediate-chain gene (DNAI1) mutations result in situs inversus and primary ciliary dyskinesia (Kartagener syndrome). *Am J Hum Genet* **68**, 1030–1035 (2001).

142. Pennarun, G. *et al.* Loss-of-Function Mutations in a Human Gene Related to Chlamydomonas reinhardtii Dynein IC78 Result in Primary Ciliary Dyskinesia. *The American Journal of Human Genetics* **65**, 1508–1519 (1999).

143. Loges, N. T. *et al.* DNAI2 Mutations Cause Primary Ciliary Dyskinesia with Defects in the Outer Dynein Arm. *Am J Hum Genet* **83**, 547–558 (2008).

144. Al-Mutairi, D. A., Alsabah, B. H., Alkhaledi, B. A., Pennekamp, P. & Omran, H. Identification of a novel founder variant in DNAI2 cause primary ciliary dyskinesia in five consanguineous families derived from a single tribe descendant of Arabian Peninsula. *Front Genet* **13**, 1–14 (2022).

145. Rocca, M. S. *et al.* A novel genetic variant in DNAI2 detected by custom gene panel in a newborn with Primary Ciliary Dyskinesia: case report. *BMC Med Genet* **21**, 1–6 (2020).

146. Young, S. A. M. *et al.* CRISPR/Cas9-mediated rapid generation of multiple mouse lines identified Ccdc63 as essential for spermiogenesis. *Int J Mol Sci* **16**, 24732–24750 (2015).

147. Knowles, M. R. *et al.* Exome sequencing identifies mutations in CCDC114 as a cause of primary ciliary dyskinesia. *Am J Hum Genet* **92**, 99–106 (2013).

148. Onoufriadis, A. *et al.* Splice-site mutations in the axonemal outer dynein arm docking complex gene CCDC114 cause primary ciliary dyskinesia. *Am J Hum Genet* **92**, 88–98 (2013).

149. Gao, Y. *et al.* Case Report: Novel Biallelic Mutations in ARMC4 Cause Primary Ciliary Dyskinesia and Male Infertility in a Chinese Family. *Front Genet* **12**, 1–7 (2021).

150. Hjeij, R. *et al.* ARMC4 mutations cause primary ciliary dyskinesia with randomization of left/right body asymmetry. *Am J Hum Genet* **93**, 357–367 (2013).

151. Onoufriadis, A. *et al.* Combined exome and whole-genome sequencing identifies mutations in ARMC4 as a cause of primary ciliary dyskinesia with defects in the outer dynein arm. *J Med Genet* **51**, 61–67 (2014).

152. Chiani, F. *et al.* Functional loss of Ccdc151 leads to hydrocephalus in a mouse model of primary ciliary dyskinesia. *DMM Disease Models and Mechanisms* **12**, (2019).

153. Wang, R. *et al.* Case Report: Identification of a Novel ODAD3 Variant in a Patient With Primary Ciliary Dyskinesia. *Front Genet* **12**, 1–5 (2021).

154. Hjeij, R. *et al.* CCDC151 mutations cause primary ciliary dyskinesia by disruption of the outer dynein arm docking complex formation. *Am J Hum Genet* **95**, 257–274 (2014).

155. Alsaadi, M. M. *et al.* Nonsense mutation in coiled-coil domain containing 151 gene (CCDC151) causes primary ciliary dyskinesia. *Hum Mutat* **35**, 1446–1448 (2014).

156. Wallmeier, J. *et al.* TTC25 Deficiency Results in Defects of the Outer Dynein Arm Docking Machinery and Primary Ciliary Dyskinesia with Left-Right Body Asymmetry Randomization. *Am J Hum Genet* **99**, 460–469 (2016).

157. Backman, K. *et al.* A splice site and copy number variant responsible for TTC25-related primary ciliary dyskinesia. *Eur J Med Genet* **64**, (2021).

158. Sasaki, K. *et al.* Calaxin is required for cilia-driven determination of vertebrate laterality. *Commun Biol* **2**, 226 (2019).

159. Hjeij, R. *et al.* Pathogenic variants in CLXN encoding the outer dynein arm docking–associated calcium-binding protein calaxin cause primary ciliary dyskinesia. *Genetics in Medicine* **25**, (2023).

160. Ma, A. *et al.* Loss-of-function mutations in CFAP57 cause multiple morphological abnormalities of the flagella in humans and mice. *JCI Insight* **8**, 1–13 (2023).

161. Bustamante-Marin, X. M. *et al.* Mutation of CFAP57, a protein required for the asymmetric targeting of a subset of inner dynein arms in Chlamydomonas, causes primary ciliary dyskinesia. *PLoS Genet* **16**, 1–27 (2020).

162. Hwang, J. Y. *et al.* Genetic Defects in DNAH2 Underlie Male Infertility With Multiple Morphological Abnormalities of the Sperm Flagella in Humans and Mice. *Front Cell Dev Biol* **9**, 1–16 (2021).

163. Gao, Y. *et al.* Novel bi-allelic variants in DNAH2 cause severe asthenoteratozoospermia with multiple morphological abnormalities of the flagella. *Reprod Biomed Online* **42**, 963–972 (2021).

164. Li, Y. *et al.* DNAH2 is a novel candidate gene associated with multiple morphological abnormalities of the sperm flagella. *Clin Genet* **95**, 590–600 (2019).

165. Zhang, Y. *et al.* Vertebrate dynein-f depends on Wdr78 for axonemal localization and is essential for ciliary beat. *J Mol Cell Biol* **11**, 383–394 (2019).

166. Tu, C. *et al.* Bi-allelic mutations of DNAH10 cause primary male infertility with asthenoteratozoospermia in humans and mice. *Am J Hum Genet* **108**, 1466–1477 (2021).

167. Li, K. *et al.* Bi-allelic variants in DNAH10 cause asthenoteratozoospermia and male infertility. *J Assist Reprod Genet* **39**, 251–259 (2022).

168. Wang, R. *et al.* Dynein axonemal heavy chain 10 deficiency causes primary ciliary dyskinesia in humans and mice. *Front Med* **17**, 957–971 (2023).

169. Wang, Y., Troutwine, B. R., Zhang, H. & Gray, R. S. The axonemal dynein heavy chain 10 gene is essential for monocilia motility and spine alignment in zebrafish. *Dev Biol* **482**, 82–90 (2022).

170. Lu, S. *et al.* Bi-allelic variants in human WDR63 cause male infertility via abnormal inner dynein arms assembly. *Cell Discov* **7**, (2021).

171. Hofmeister, W. *et al.* Targeted copy number screening highlights an intragenic deletion of WDR63 as the likely cause of human occipital encephalocele and abnormal CNS development in zebrafish. *Hum Mutat* **39**, 495–505 (2018).

172. King, A. *et al.* Dynll1 is essential for development and promotes endochondral bone formation by regulating intraflagellar dynein function in primary cilia. **28**, 2573–2588 (2019).

173. Schmidts, M. *et al.* TCTEX1D2 mutations underlie Jeune asphyxiating thoracic dystrophy with impaired retrograde intraflagellar transport. *Nat Commun* **6**, (2015).

174. Tang, S. *et al.* Biallelic Mutations in CFAP43 and CFAP44 Cause Male Infertility with Multiple Morphological Abnormalities of the Sperm Flagella. *Am J Hum Genet* **100**, 854–864 (2017).

175. Sha, Y. W. *et al.* Novel Mutations in CFAP44 and CFAP43 Cause Multiple Morphological Abnormalities of the Sperm Flagella (MMAF). *Reproductive Sciences* **26**, 26–34 (2019).

176. Wu, H. *et al.* NovelCFAP43 andCFAP44 mutations cause male infertility with multiple morphological abnormalities of the sperm flagella (MMAF). *Reprod Biomed Online* (2019) doi:10.1016/j.rbmo.2018.12.037.

177. Coutton, C. *et al.* Mutations in CFAP43 and CFAP44 cause male infertility and flagellum defects in Trypanosoma and human. *Nat Commun* **9**, (2018).

178. Morimoto, Y. *et al.* Nonsense mutation in CFAP43 causes normal-pressure hydrocephalus with ciliary abnormalities. *Neurology* **92**, E2364–E2374 (2019).

179. Ben Khelifa, M. *et al.* Mutations in DNAH1, which encodes an inner arm heavy chain dynein, lead to male infertility from multiple morphological abnormalities of the sperm flagella. *Am J Hum Genet* **94**, 95–104 (2014).

180. Amiri-Yekta, A. *et al.* Whole-exome sequencing of familial cases of multiple morphological abnormalities of the sperm flagella (MMAF) reveals new DNAH1 mutations. *Human Reproduction* **31**, 2872–2880 (2016).

181. Wang, X. *et al.* Homozygous DNAH1 frameshift mutation causes multiple morphological anomalies of the sperm flagella in Chinese. *Clin Genet* **91**, 313–321 (2017).

182. Sha, Y. *et al.* DNAH1 gene mutations and their potential association with dysplasia of the sperm fibrous sheath and infertility in the Han Chinese population. *Fertil Steril* **107**, 1312-1318.e2 (2017).

183. Neesen, J. *et al.* Disruption of an inner arm dynein heavy chain gene results in asthenozoospermia and reduced ciliary beat frequency. *Hum Mol Genet* **10**, 1117–1128 (2001).

184. Liu, M. *et al.* Successful Live Birth following Natural Cycle Oocyte Retrieval in a Woman with Primary Infertility and Atypical Primary Ovarian Insufficiency with a DNAH1 Gene Deletion Mutation. *Genet Test Mol Biomarkers* **25**, 668–673 (2021).

185. Imtiaz, F., Allam, R., Ramzan, K. & Al-Sayed, M. Variation in DNAH1 may contribute to primary ciliary dyskinesia. *BMC Med Genet* **16**, 1–6 (2015).

186. Emiralioğlu, N. *et al.* Genotype and phenotype evaluation of patients with primary ciliary dyskinesia: First results from Turkey. *Pediatr Pulmonol* **55**, 383–393 (2020).

187. Yuan, L. *et al.* Identification of novel compound heterozygous variants in the DNAH1 gene of a Chinese family with left-right asymmetry disorder. *Front Mol Biosci* **10**, (2023).

188. Meng, G. Q. *et al.* Bi-allelic variants in DNAH3 cause male infertility with asthenoteratozoospermia in humans and mice. *Hum Reprod Open* **2024**, (2024).

189. Shen, Y. *et al.* Loss-of-function mutations in QRICH2 cause male infertility with multiple morphological abnormalities of the sperm flagella. *Nat Commun* **10**, 1–15 (2019).

190. Huang, F. *et al.* A novel frameshift mutation in DNAH6 associated with male infertility and asthenoteratozoospermia. *Front Endocrinol (Lausanne)* **14**, 1–9 (2023).

191. Li, Y. *et al.* DNAH6 and Its Interactions with PCD Genes in Heterotaxy and Primary Ciliary Dyskinesia. *PLoS Genet* **12**, 1–20 (2016).

192. Gao, Y. *et al.* Loss of function mutation in DNAH7 induces male infertility associated with abnormalities of the sperm flagella and mitochondria in human. *Clin Genet* **102**, 130–135 (2022).

193. Wei, X. *et al.* Bi-allelic mutations in DNAH7 cause asthenozoospermia by impairing the integrality of axoneme structure. *Acta Biochim Biophys Sin (Shanghai)* **53**, 1300–1309 (2021).

194. Zhang, Y. J. *et al.* Identification of dynein heavy chain 7 as an inner arm component of human cilia that is synthesized but not assembled in a case of primary ciliary dyskinesia. *Journal of Biological Chemistry* **277**, 17906–17915 (2002).

195. Li, Y. *et al.* Whole-exome sequencing of a cohort of infertile men reveals novel causative genes in teratozoospermia that are chiefly related to sperm head defects. *Human Reproduction* **37**, 152–177 (2022).

196. Oud, M. S. *et al.* Exome sequencing reveals variants in known and novel candidate genes for severe sperm motility disorders. *Human Reproduction* **36**, 2597–2611 (2021).

197. Yang, M. *et al.* Deficiency in a special dynein DNAH12 causes male infertility by impairing DNAH1 and DNALI1 recruitment in humans and mice. *Elife* (2024).

198. Sha, Y. *et al.* Homozygous mutation in DNALI1 leads to asthenoteratozoospermia by affecting the inner dynein arms. *Front Endocrinol (Lausanne)* **13**, 1–8 (2023).

199. Zhang, F. *et al.* Splicing Mutation in DNALI1 Causes Male Infertility with Severe Oligoasthenoteratozoospermia in Humans. *Reproductive Sciences* **31**, 1610–1616 (2024).

200. Wu, H. *et al.* DNALI1 deficiency causes male infertility with severe asthenozoospermia in humans and mice by disrupting the assembly of the flagellar inner dynein arms and fibrous sheath. *Cell Death Dis* **14**, (2023).

201. Zhou, Y. *et al.* Dnali1 is required for sperm motility and male fertility in mice. *Basic Clin Androl* **33**, 1–11 (2023).

202. Yap, Y. T. *et al.* DNALI1 interacts with the MEIG1/PACRG complex within the manchette and is required for proper sperm flagellum assembly in mice. *Elife* **12**, 1–27 (2023).

203. Mazor, M. *et al.* Primary ciliary dyskinesia caused by homozygous mutation in DNAL1, encoding dynein light chain 1. *Am J Hum Genet* **88**, 599–607 (2011).

204. Sudhakar, D. V. S. *et al.* Exome sequencing and functional analyses revealed CETN1 variants leads to impaired cell division and male fertility. *Hum Mol Genet* **32**, 533–542 (2023).

205. Avasthi, P. *et al.* Germline deletion of cetn1 causes infertility in male mice. *J Cell Sci* **126**, 3204–3213 (2013).

206. Lorès, P. *et al.* Mutations in TTC29, Encoding an Evolutionarily Conserved Axonemal Protein, Result in Asthenozoospermia and Male Infertility. *Am J Hum Genet* **105**, 1148–1167 (2019).

207. Liu, C. *et al.* Bi-allelic Mutations in TTC29 Cause Male Subfertility with Asthenoteratospermia in Humans and Mice. *Am J Hum Genet* **105**, 1168–1181 (2019).

208. Dacheux, D. *et al.* Novel axonemal protein ZMYND12 interacts with TTC29 and DNAH1, and is required for male fertility and flagellum function. *Elife* **12**, 1–25 (2023).

209. Kaye, E. G. *et al.* RNA polymerase II pausing is essential during spermatogenesis for appropriate gene expression and completion of meiosis. *Nat Commun* **15**, 1–17 (2024).

210. Walton, T. *et al.* Axonemal structures reveal mechanoregulatory and disease mechanisms. *Nature* **618**, 625–633 (2023).
