# Supplementary material for: Structural diversity of axonemes across mammalian motile cilia
Source: Nature. 2025 Jan 1;637(8048):1170–7. doi: 10.1038/s41586-024-08337-5 (PMC11779644; doi:10.1038/s41586-024-08337-5)
Supplement: Supplementary file 2 — Reporting Summary [file 41586_2024_8337_MOESM2_ESM.pdf]

nature portfolio

Corresponding author(s): Alan Brown, Tzviya Zeev-Ben-Mordehai, Rui Zhang

Last updated by author(s): Oct 31, 2024

Reporting Summary

Nature Portfolio wishes to improve the reproducibility of the work that we publish. This form provides structure for consistency and transparency in reporting. For further information on Nature Portfolio policies, see our Editorial Policies and the Editorial Policy Checklist.

Statistics

For all statistical analyses, confirm that the following items are present in the figure legend, table legend, main text, or Methods section.

n/a	Confirmed	
☒	☐	The exact sample size (n) for each experimental group/condition, given as a discrete number and unit of measurement
☒	☐	A statement on whether measurements were taken from distinct samples or whether the same sample was measured repeatedly
☒	☐	The statistical test(s) used AND whether they are one- or two-sided<br>*Only common tests should be described solely by name; describe more complex techniques in the Methods section.*
☒	☐	A description of all covariates tested
☒	☐	A description of any assumptions or corrections, such as tests of normality and adjustment for multiple comparisons
☒	☐	A full description of the statistical parameters including central tendency (e.g. means) or other basic estimates (e.g. regression coefficient) AND variation (e.g. standard deviation) or associated estimates of uncertainty (e.g. confidence intervals)
☒	☐	For null hypothesis testing, the test statistic (e.g. F, t, r) with confidence intervals, effect sizes, degrees of freedom and P value noted<br>*Give P values as exact values whenever suitable.*
☒	☐	For Bayesian analysis, information on the choice of priors and Markov chain Monte Carlo settings
☒	☐	For hierarchical and complex designs, identification of the appropriate level for tests and full reporting of outcomes
☒	☐	Estimates of effect sizes (e.g. Cohen's d, Pearson's r), indicating how they were calculated

Our web collection on statistics for biologists contains articles on many of the points above.

Software and code

Policy information about availability of computer code

Data collection	SerialEM v3.8, SerialEM v4.0, Warp v1.0.9
Data analysis	cryoSPARC v3.4.0, FREALIGN v9.11, RELION v3.1, RELION v4.0.1, deepEMhancer (no version number), Chimera v1.15, ChimeraX v1.4, 1.5, or 1.6, IMOD v4.12.37, PEET v1.17.0, SWISS-MODEL (no version number), AlphaFold2, Coot v0.9.8, Phenix v1.19 or 1.20, Namdinator (no version number), ModelAngelo v1.0, findMySequence v0.8.6, DALI server (no version number), DeepTracerID (no version number), FoldSeek (no version number), MOLREP v11, SITUS v3.1, Mascot v2.7.0, Sequest v28.13, custom scripts available from https://github.com/rui--zhang/Doublet

For manuscripts utilizing custom algorithms or software that are central to the research but not yet described in published literature, software must be made available to editors and reviewers. We strongly encourage code deposition in a community repository (e.g. GitHub). See the Nature Portfolio guidelines for submitting code & software for further information.

Data

Policy information about availability of data

All manuscripts must include a data availability statement. This statement should provide the following information, where applicable:

- Accession codes, unique identifiers, or web links for publicly available datasets
- A description of any restrictions on data availability
- For clinical datasets or third party data, please ensure that the statement adheres to our policy

Composite cryo-EM maps of the 96-nm repeat of axonemal DMTs from bovine sperm, bovine oviductal cilia, human oviductal cilia, and porcine brain ventricle cilia have been deposited to the EMDB with codes EMD-50664, EMD-45783, EMD-45785, and EMD-45784 respectively. Local refinements for bovine sperm have been deposited to the EMDB with codes EMD-50866 to -50886; for bovine oviduct with codes EMD-45683 to -45697; for human oviduct with codes EMD-45714 to -45725 and -45790; and for porcine brain ventricle with codes EMD-45699 to -45713. Subtomogram averages of the 96-nm repeat of axonemal DMTs from porcine oviductal cilia have been deposited with codes EMD-45677 to -45680. Cryo-EM maps of the 48-nm DMT repeat from bovine oviductal cilia and porcine brain ventricle cilia have been deposited with codes EMD-45801 and -45802. The atomic model of the 96-nm repeat of the bovine sperm DMT is has been deposited to the PDB with accession code 9FQR. Atomic models of the 48-nm DMT repeat from bovine oviductal cilia and porcine brain ventricle cilia have been deposited to the PDB with accession codes 9CPB and 9CPC respectively. Previously-reported atomic models of the 96-nm repeat from human respiratory cilia and of the 48-nm repeat from bovine sperm were used as initial models and are available with PDB accession codes 8J07 and 8OTZ respectively. Proteomics data from bovine oviductal cilia, human oviductal cilia, and porcine brain ventricle cilia are available in Supplementary Table 4. Custom scripts used in this study are publicly available at https://github.com/rui--zhang/Doublet.

Research involving human participants, their data, or biological material

Policy information about studies with human participants or human data. See also policy information about sex, gender (identity/presentation), and sexual orientation and race, ethnicity and racism.

Reporting on sex and gender	Oviduct samples were provided by female organ donors.
Reporting on race, ethnicity, or other socially relevant groupings	Data on race, ethnicity and social groupings was not shared with us during procurement or processing of the tissue.
Population characteristics	Age, genotypic information and medical history were not shared with us during procurement or processing of the tissue.
Recruitment	Participants were registered organ donors.
Ethics oversight	The protocol of procurement and processing of human uteri was reviewed by an Institutional Review Board of Harvard University (Protocol# IRB21-0272), which determined the tissue procurement and processing was not human subject research. No identifying information of the deceased organ donors was shared in procurement or processing of the tissue.

Note that full information on the approval of the study protocol must also be provided in the manuscript.

Field-specific reporting

Please select the one below that is the best fit for your research. If you are not sure, read the appropriate sections before making your selection.

☒ Life sciences ☐ Behavioural & social sciences ☐ Ecological, evolutionary & environmental sciences

For a reference copy of the document with all sections, see nature.com/documents/nr-reporting-summary-flat.pdf

Life sciences study design

All studies must disclose on these points even when the disclosure is negative.

Sample size	For cryo-EM/ET processing, no methods were used to predetermine sample size. The size of the cryo-EM datasets was determined by the need to obtain structures with sufficient resolution to identify differences, and for the bovine sperm doublet microtubule, to build an atomic model. The number of micrographs and particles are listed in the Extended Data.
Data exclusions	Micrographs with low resolution estimates following CTF fitting were discarded. Some tilt images were discarded from tomogram reconstruction. The algorithms used for image processing may down-weigh or exclude particles as part of their refinement strategy.
Replication	Replication is not necessary for structural studies. Cryo-EM maps represent an average of many thousands of individual copies of the complex of interest, collected from multiple preparations across several microscope sessions. Structures of oviductal cilia doublet microtubules were obtained from three different organisms (bovine, porcine, and human).
Randomization	For calculation of the Fourier Shell Correlation (FSC), cryo-EM particles or pseudosubtomograms were randomly split into two halves.
Blinding	Blinding is not necessary since there are no groups that need subjective analysis.

Reporting for specific materials, systems and methods

We require information from authors about some types of materials, experimental systems and methods used in many studies. Here, indicate whether each material, system or method listed is relevant to your study. If you are not sure if a list item applies to your research, read the appropriate section before selecting a response.

Materials & experimental systems

n/a	Involved in the study
☒	☐ Antibodies
☒	☐ Eukaryotic cell lines
☒	☐ Palaeontology and archaeology
☐	☒ Animals and other organisms
☒	☐ Clinical data
☒	☐ Dual use research of concern
☒	☐ Plants

Methods

n/a	Involved in the study
☒	☐ ChIP-seq
☒	☐ Flow cytometry
☒	☐ MRI-based neuroimaging

Animals and other research organisms

Policy information about studies involving animals; ARRIVE guidelines recommended for reporting animal research, and Sex and Gender in Research

Laboratory animals	The study did not involve laboratory animals.
Wild animals	The study did not involve wild animals.
Reporting on sex	Sperm samples were obtained from male animals. Oviduct samples were obtained from female animals. The sex of the animals of the brain samples is unknown.
Field-collected samples	The study did not involve samples collected from the field.
Ethics oversight	No ethical approval or guidance was required because organs were used from animals sacrificed for other purposes. Porcine oviductal tissue was acquired from Animal Technologies. Bovine oviducts and porcine brains were sourced from Trenton Processing Center (Trenton, IL) or the Division of Comparative Medicine at Washington University in St. Louis.

Note that full information on the approval of the study protocol must also be provided in the manuscript.

Plants

Seed stocks	n/a
Novel plant genotypes	n/a
Authentication	n/a
